# 2-DE analysis indicates that *Acinetobacter baumannii *displays a robust and versatile metabolism

**DOI:** 10.1186/1477-5956-7-37

**Published:** 2009-09-28

**Authors:** Nelson C Soares, Maria P Cabral, José R Parreira, Carmen Gayoso, Maria J Barba, Germán Bou

**Affiliations:** 1 Servicio de Microbiologia-Unidad de Investigacion, Complejo Hospitalario Universitario A Coruña, 15006 La Coruña, Spain

## Abstract

**Background:**

*Acinetobacter baumannii *is a nosocomial pathogen that has been associated with outbreak infections in hospitals. Despite increasing awareness about this bacterium, its proteome remains poorly characterised, however recently the complete genome of *A. baumannii *reference strain ATCC 17978 has been sequenced. Here, we have used 2-DE and MALDI-TOF/TOF approach to characterise the proteome of this strain.

**Results:**

The membrane and cytoplasmatic protein extracts were analysed separately, these analyses revealed the reproducible presence of 239 and 511 membrane and cytoplamatic protein spots, respectively. MALDI-TOF/TOF characterisation identified a total of 192 protein spots (37 membrane and 155 cytoplasmatic) and revealed that the identified membrane proteins were mainly transport-related proteins, whereas the cytoplasmatic proteins were of diverse nature, although mainly related to metabolic processes.

**Conclusion:**

This work indicates that *A. baumannii *has a versatile and robust metabolism and also reveal a number of proteins that may play a key role in the mechanism of drug resistance and virulence. The data obtained complements earlier reports of *A. baumannii *proteome and provides new tools to increase our knowledge on the protein expression profile of this pathogen.

## Background

*Acinetobacter baumannii *is a Gram-negative, nonmotile, aerobic coccobacillus that is often found in health care settings. There is increasing concern as regarding the prevalence of this bacterium in hospital environments, especially in intensive care units, where it has been associated with different types of infections such as pneumonia, meningitis, bacteraemia, urinary tract infections and others [1-3]. In addition, *A. baumannii *has a remarkable capacity to acquire resistance to most antibiotics used in clinical practice and to cause outbreaks throughout cities, countries and even continents [4-6]. Despite the emerging awareness of the potential hazards of this bacterium, little is known about the mechanisms that operate during antibiotic resistance, virulence, or persistence strategies of *A. baumannii*.

Genomic approaches have recently been successfully applied for *A. baumannii*. For instance, genomic comparison between SDF and AYE strains with the soil-living *A. baylyi *strain ADP1 revealed important exclusive features of each strain, which may partly explain their existence in different ecological niches [7,8]. The recently described sequence of *A. baumannii *ATCC 17978 isolate revealed that its genome is composed by 3,976,746 base pairs (bp) and that it has 3,830 open reading frames (ORFs), of which nearly 17% are situated in 28 putative alien islands [9], which suggests that the genome has acquired a significant amount of foreign DNA.

Nevertheless, it is widely accepted that most ORFs are expressed under different conditions and/or states of growth and environmental stress [10]. Moreover, the identification of proteins via MS/MS analyses validate predicted genes, correct erroneous gene annotation and reveal some completely missed genes [10,11]. Therefore as pointed out by Brötz-Oesterhelt *et al. *(2005) [12], the application of further techniques such as proteomics is required in order to establish the protein composition of a given cell under certain conditions, independently of its linear gene sequence.

Proteomics analysis has been applied to fractions enriched with *A. baumannii *cell membranes, and a group of 22 proteins, composed by ribosomal proteins, chaperones elongation factors and outer membrane proteins identified [13]. A total of 135 proteins (inner and outer membrane) and 23 periplasmic proteins were identified in *A. baumannii *in another study [14]. More recently, it was characterise the proteome of outer membrane vesicles from a clinical *A. baumannii *isolate [15]. These studies provide important data regarding the proteome of *Acinectobacter*, nevertheless as far as we are aware, the cytoplasmatic proteome of *A. baumannii *remains to be characterised. In the current study, we used 2-DE and MALDI-TOF/TOF to analyse the membrane and cytoplasmatic proteome of *A. baumannii*, and the data obtained confirms the results of previous genomic studies showing that *A. baumannii *displays a robust and versatile metabolism capable of exploiting a variety of carbon sources and energy.

## Materials and methods

### Bacterial strain and growth conditions

*A. baumannii *ATCC 17978 was grown overnight in Mueller Hinton broth (Fluka, Madrid, Spain) at 37°C under constant shaking. Fresh nutrient media (500 mL) was inoculated with a 1:100 dilution of the overnight culture and grown to OD_600 _= 0.4-0.6, at 37°C with vigorous shaking.

### Protein extraction

The cell were harvested by centrifugation at 3,500 g for 15 min at 4°C and washed twice with 10 mL 0.9% (w/v) NaCl. The resultant pellet was resuspended in 3 or 5 mL of disintegration buffer [13] (7.8 g/L NaH_2_PO_4_, 7.1 g/L Na_2_HPO_4_, 0.247 g/L MgSO4 7.H_2_O + protease inhibitor mix (GE Healthcare, USA) + nuclease mix (GE Healthcare, USA)) and sonicated on ice for 3 periods of 5 min. The unbroken cells were separated by centrifugation at 1,500 *g*. The supernatant was centrifuged for 30 min at 4°C at 4,500 rpm and was then clarified through a 0.45 μM filter (Milipore, USA) to remove the cell debris. Finally, the extract was processed with a 2-DE Cleanup Kit (GE Healthcare, USA).

The same extraction method was used to extract cell surface membrane. However, after the separation of unbroken cells, the lysate was treated as described by Molly *et al. *(2000) [16]. Briefly, an equal volume of ice-cold 0.1 M sodium carbonate (pH 11) was added to the resulting supernatant and the mixture was stirred slowly overnight, on ice. The carbonate treated membranes were then collected by ultracentrifugation at 100,000 *g *for 45 min at 4°C, and the membranes were then re-suspended in 500 μl H_2_O. Finally, as with the soluble fraction, the extract was processed with a 2-DE Cleanup Kit (GE Healthcare, USA).

### Two-dimensional gel electrophoresis (2-DE)

Protein concentration in the extracts was determined with a Biorad protein assay kit (Biorad, Germany), by a modified Bradford assay [17] as suggested by Ramagli *et al. *(1999) [18].

For isoelectric focusing (IEF), the IPGphor III system was used (GE Healthcare, USA) with 3-10 non-linear (NL), 4-7 (L) or 6-11(L) pH gradient strips (IPG strips, GE Healthcare, Sweden). Proteins were solubilised in 8 M urea, 2% (w/v) CHAPS, 40 mM DTT and 0.5% (v/v) corresponding IPG buffer (GE Healthcare, Sweden). IEF was carried out at 30 V for 12 h, followed by 250 V for 1 h, 500 V for 1.5 h, 1,000 V for 1.5 h, a gradient to 8,000 V over 1.5 h and maintenance at 8,000 V for a further 4 h, all at 20°C. Note, for 6-11 (L) pH gradient strips, IEF was carried out at 30 V for 12 h, followed by 500 V for 1 h, 1,000 V for 1 h, a gradient to 8,000 V for 2.5 h and maintenance at 8,000 V for 0.5 h. Prior to the second dimension (SDS-PAGE), the focused IPG strips were equilibrated for 2 × 15 min in buffer containing 50 mM Tris-HCl (pH 8.8), 6 M urea, 30% (v/v) glycerol, 2% (w/v) SDS and a trace of Bromophenol Blue. DTT at 1% (w/v) was added to the first equilibration step and 2.5% (w/v) iodoacetamide to the second. SDS-PAGE was performed on 12% or 15% polyacrylamide gels [19]. For analytical 2-DE gels, silver staining was performed according to Blum *et al. *(1987) [20] and gels were loaded with 25 to 40 μg of total protein. For preparative gels, MS-compatible silver staining [21] was used and the gels were loaded with at least 350 μg of total protein.

### Image acquisition and 2-DE analyses

Gels were scanned with an Image Scanner v3.3 densitometer (GE Healthcare) and analysed with Image Master Platinum software, V.6.0, as described by the manufacturer (GE Healthcare). Briefly, a matched set consisting of three images was created. In order to confirm reproducibility, at least three biological replicates were processed. For spot detection, the parameters were adjusted in the following order: smooth 2; MinArea 5; Saliency 1.00000, and only spots observed on all three gels of a replicated group were considered for further analyses. Numerical data for individual spots were generated automatically and expressed as % of spot volume.

### Trypsin digestion of proteins and characterisation by MALDI-TOF/TOF

Selected spots were manual excised from the gels and transferred to microcentrifuge tubes. Samples were then destained with a solution containing 20% (w/v) sodium thiosulphate and 1% (w/v) potassium ferricyanide for 5 min. Destained spots were then in-gel reduced, alkylated and digested with trypsin as suggested be Sechi and Chait 1998 [22]. Briefly, spots were washed twice with 25 mM ammonium bicarbonate in 50% (v/v) acetonitrile (ACN) for 20 min. The gel spots were then shrunk with 100% (v/v) in acetonitrile and dried in a speed-vac (Savant, USA). The samples were reduced with DTT and subsequently alkylated with iodoacetamide. Samples were digested with 20 ng/μl sequencing grade trypsin (Roche Applied Science, USA), overnight at 37°C. After digestion the supernatant was collected and 1 μl was spotted onto a MALDI target plate (384-spot Teflon^®^-coated plates) and allowed to air dry at room temperature. Where necessary, tryptic peptides were passed through C18 Zip-Tips (Millipore, USA) and mixed with 3 mg/ml of an α-cyano-4-hydroxycinnamic acid in 0.1% (v/v) TFA and 50% (v/v) ACN was added to the dried peptide digest spots and allowed to air dry. The samples were analyzed using a MALDI-TOF/TOF mass spectrometer 4800 Proteomics Analyzer (Applied Biosystems). MALDI-TOF spectra were acquired in reflector positive ion mode using 1000 laser shots per spectrum. Data Explorer version 4.2 (Applied Biosystems) was used for spectra analyses and generating peak picking list. All mass spectra were internally calibrated using autoproteolytic trypsin fragments and externally calibrate using standard peptide mixture (Sigma-Aldrich). TOF/TOF fragmentation spectra were acquired by selecting the 10 most abundant ions of each MALDI-TOF peptide mass map (excluding trypsin autolytic peptides and other background ions) and averaging 2,000 laser shots per fragmentation spectrum. The parameters used to analyze the data were a signal to noise threshold of 20, minimum area of 100 and a resolution higher than 10,000 with a mass accuracy of 20 ppm.

### Database queries and protein identification

The monoisotopic peptide mass fingerprinting data obtained from MS and the amino acid sequence tag obtained from each peptide fragmentation in MS/MS analyses were used to search for protein candidates using Mascot version 1.9 from Matrix Science . Peak intensity was used to select up to 50 peaks per spot for peptide mass fingerprinting and 50 peaks per precursor for MS/MS identification. Tryptic autolytic fragment-, keratin-, and matrix-derived peaks were removed from the data set utilised for databased search. The search for peptides mass fingerprints and tandem MS spectra were performed in NCBInr database without any taxonomy restriction. Fixed and variable modifications were considered (Cys as S-carbadiomethyl and Met as oxidised methionine, respectively), allowing one trypsin missed cleavage. MS/MS ions search were conducted with a mass tolerance of ± 1.2 Da on the parent and 0.3-0.8 Da on fragments, in all cases the peptide charge was +1. Decoy search was done automatically by Mascot on randomized database of equal composition and size. Mascot scores for all protein identifications were higher than the accepted threshold for significance (at the *p *< 0.050 level, positive rate measured to be 0.047). For protein subcellular localisation prediction it was utilised PSORTb v.2.0.4, available free online at .

## Results and Discussion

### Bimodal distribution of Acinetobacter baumannii proteome

In the current study, a 2-DE based proteomic analyses of *A. baumannii *ATCC 17978, grown under rich medium conditions and harvested during exponential phase were performed. The study was based on the analysis of two distinct protein fractions, a fraction enriched in membrane proteins and a fraction enriched in cytoplasmatic proteins; for convenience, these are referred in the text as membrane and cytoplasmatic fractions respectively. Analysis of three biological replicates revealed that at least 239 spots were reproducibly detected in the membrane fraction, whereas the cytoplasmatic fraction contained a total of 511 reproducible spots.

**Figure 1:**
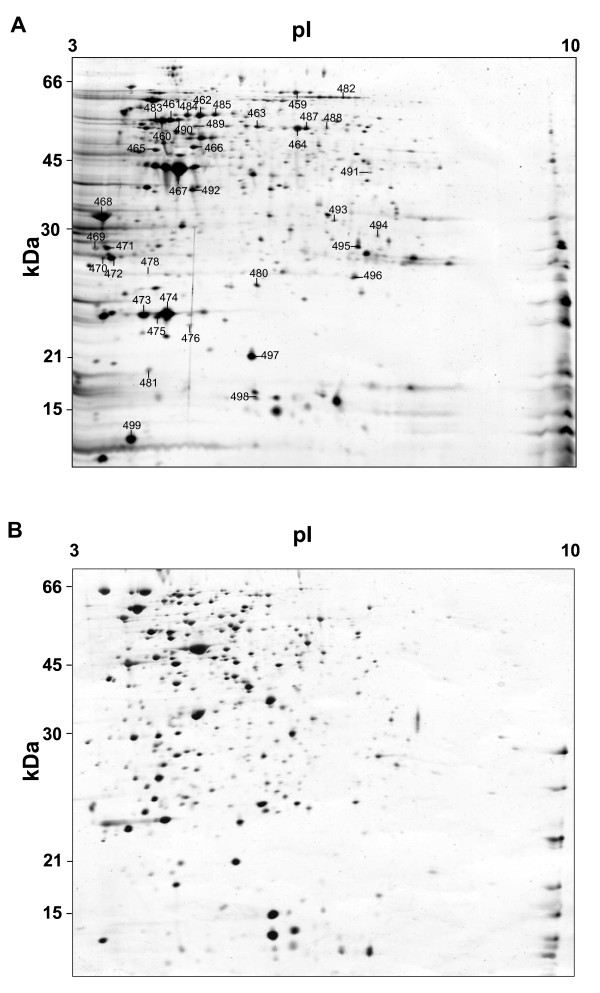
**2-DE gels showing *A. baumannii *proteins**. (A) membrane proteins, (B) cytoplasmatic proteins. Numbered spots (in A) indicate membrane proteins identified by MALDI-TOF/TOF. All gels (12% SDS) were silver stained and loaded with 25 μg total protein.

**Figure 2:**
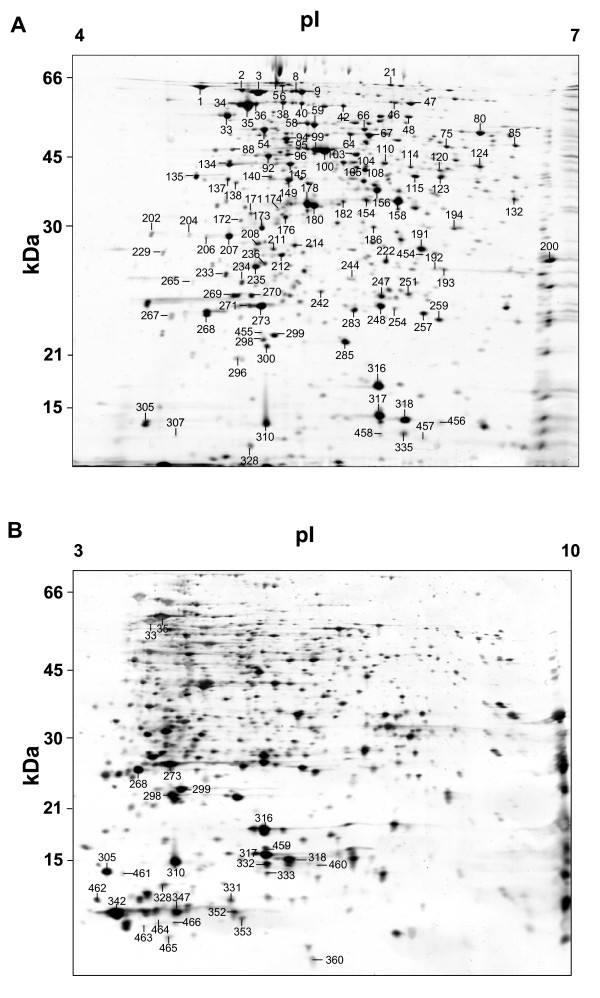
**The cytoplasmatic fraction was analysed with IPG strips of various pH range and silver stained gels with different concentrations of acrylamide**. 2-DE (12% SDS) gels containing proteins within a range pH 4-7 (A), 2-DE (15% SDS) gel containing proteins within a range pH 3-10 (B), gels were loaded with 25 μg, total protein. Numbered spots indicate proteins identified by MALDI-TOF/TOF.

**Figure 3:**
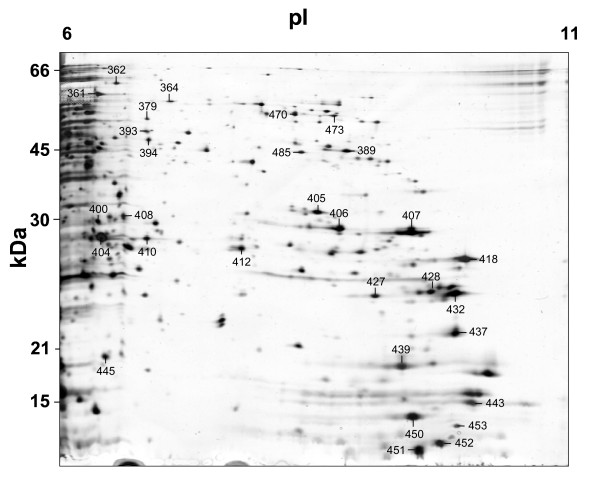
**2-DE (12% SDS) gel containing basic proteins within a pH range of 6-11**. Gel was loaded with 40 μg, total protein. Numbered spots indicate proteins identified by MALDI-TOF/TOF.

As a first approach, both the membrane and the cytoplasmatic protein fractions were analysed by use of 2-DE gels with immobilized pH gradient (IPG) strips (pH 3-10) (see Fig. 1A and 1B, respectively). However, for the cytoplasmatic fraction, these analyses revealed a clear predominance of acidic proteins, although a number of highly basic proteins remained unresolved close to the cathode (p*I* > 9) (see Fig. 1B). Therefore, in an attempt to overcome the intrinsic under-representation of the alkaline proteins [12,23], the acidic and basic cytoplasmatic sub-proteomes were analysed using immobilized pH gradient strips of various pH range (see Fig. 2 and 3). Use of 2-DE 12% polyacrylamide (p*I *ranges of 4-7) silver stained gels revealed 390 spots that could be reproducibly detected on the acidic range (Fig. 2A). Additionally, complementary analyses using 15% polyacrylamide silver stain gels in the range pH 3-10 revealed the presence of further 30 protein spots of low molecular weight (between 9 and 12 kDa) (Fig. 2B). Use of 2-DE gels 12% polyacrylamide (p*I *ranges of 6-11) added a further 91 reproducible alkaline protein spots (nearly 21% of the total soluble protein spots) (Fig. 3).

**Figure 4:**
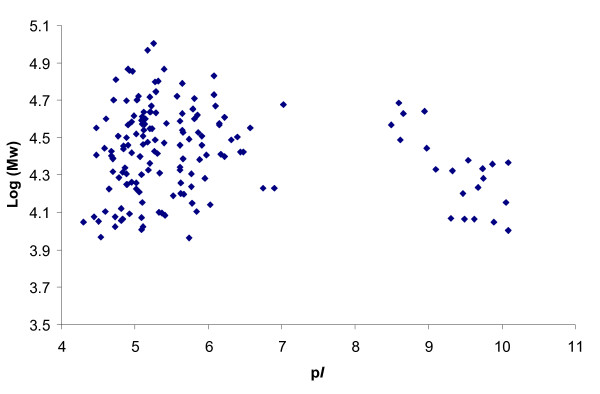
**Representation of the bimodal distribution of the all identified proteins, according to the predicted p*I *and molecular weight**. The most crowded regions are found at pH range of 4-7 and 9-11.

In concordance with previous reports referring other bacteria species [23], the 2-DE gels analysis revealed that the spot pattern of this pathogen is characterised by clear predominance of high molecular protein spots and in terms of p*I *there is visible a bimodal distribution of protein spots, where the most crowded regions is found between p*I* 4-7 and 9-11 (Fig. 4).

### Characterisation of the Acinetobacter baumannii proteome

In the current study we wished to identify the most abundant proteins spots, since these are likely to play a relevant role in bacterial biology. A total of 192 protein spots were identified 37 and 155 from membrane and cytoplasmatic protein fractions respectively. The list included proteins within a p*I* range of 4.3-10.5 and a Mw from 9.2 - 73.8 kDa (table S1, Additional file 1 and table S2, Additional file 2), indicating that the 2-DE analyses provided a representative view of the *A. baumannii *proteome. Most of the identifications were based on multiple peptide matches (table S3, Additional file 3), although in some cases protein identification was based on a single peptide sequence (namely spot # 36, 110, 134, 172, 242, 248, 459 - see table S1, Additional file 1 and table S2, Additional file 2, table S3, Additional file 3,). However, the scores for all proteins identified here were higher than the significance threshold (determined at the 95% confidence level) calculated by Mascot (table S1, Additional file 1 and table S2, Additional file 2) and in all cases the data was search against an Mascot decoy database in order to check for false positive (additional identification data for all spots is provided as supplementary material). Furthermore, all the proteins identified belong to *A. baumannii *proteome, and from the 192 spots, 172 were in fact matched with proteins belonging to *A. baumannii *ATCC 17978 proteome. This criterion, together with the fact that in most of the cases the theoretical molecular weights and/or p*I *of the identified proteins were very close to that observed in gel (table S1, Additional file 1 and table S2, Additional file 2) provided additional confidence in the identification.

Occasionally, protein spots with only small shifts in molecular weight and/or p*I *were identified with the same sequence in the data base (*e.g. *spots; 34, 35 & 36, 64 & 67; spots 94 & 95, spots 271 & 273, 469 & 472; 473 & 474 among others, see also table S1, Additional file 1 and table S2, Additional file 2). These observations are a common feature of 2-DE gels [14,24], and possibly arise from the heterogeneity in post-translation modifications. Therefore within this context, membrane and cytoplasmatic fractions together contained 153 unique proteins. Other spots shared homology with the same protein families (*e.g. *spot # 1, spot # 2 and spot # 35), but with different corresponding gene sequences, indicating the presence of multi-gene families in the *A. baumannii *proteome.

**Figure 5:**
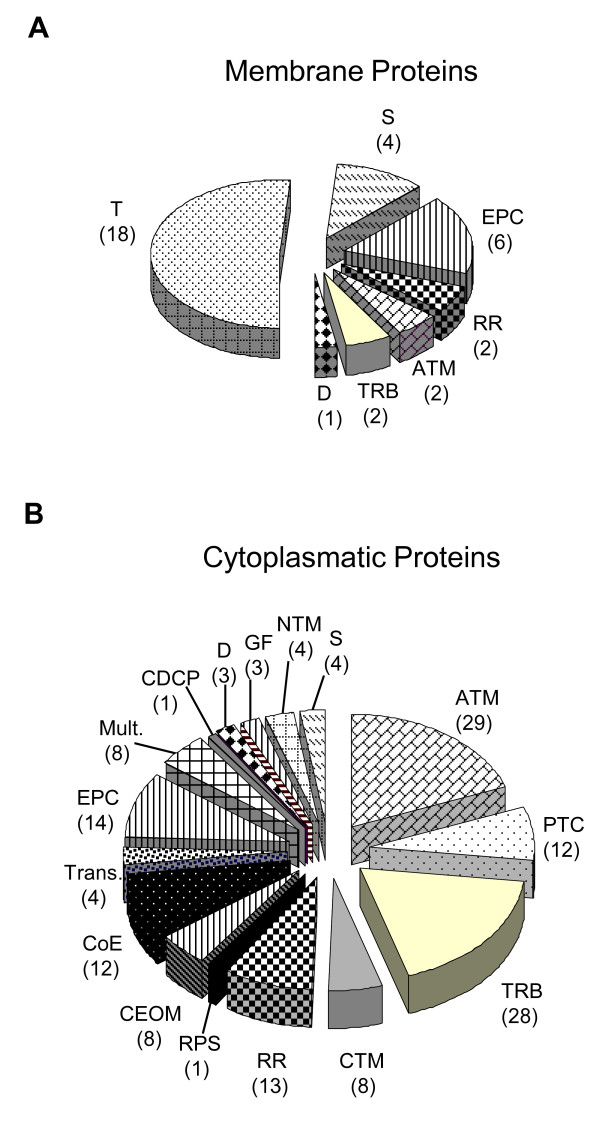
**A functional classification of the 37 most abundant protein spots in the membrane fraction (A) and of the 155 most abundant protein spots in the cytoplasmatic fraction (B)**. The protein class was abbreviated as follows: ATM, amino acid transport and metabolism; CDCP, cell division and chromosome partitioning; CTM, carbohydrate transport and metabolism; CEOM, cell envelope biogenesis and outer membrane; CoE, coenzyme metabolism; D, Defence; EPC, energy production and conversion; GF, general function predicted only; Mult., multifunctional; NTM, nucleotide transport and metabolism; PTC, postranslation modification, protein turnover, chaperones; RPS, RNA and protein synthesis; RR, proteins involved in redox reactions; S, signalling; TRB, translation, ribosomal structure and biogenesis; Trans., transcription; T, transport. Note: Hypothetical proteins (3) with unknown function are not included in this classification.

The results demonstrate that the *A. baumannii *proteome is diverse in nature, and can be conveniently divided into at least 16 distinct functional groups, namely: amino acid transport and metabolism; carbohydrate transport and metabolism; cell division and chromosome partitioning; coenzyme metabolism; cell envelope and outer membrane biogenesis (transport); defence; energy production and conversion; general function predicted only; multifunctional; nucleotide transport and metabolism; postranslation modification, protein turnover, chaperones; redox reactions; RNA and protein synthesis; signalling; translation, ribosomal structure and biogenesis and transcription (see Fig. 5), in addition there was further 4 hypothetical proteins with unknown function. Whenever possible the proteins were classified according to the clusters of orthologous groups (COG) [25].

### Membrane extract

MALDI-TOF/TOF analyses of 37 selected membrane spots revealed that 12 were identified as outer membrane proteins (Omps) (*e.g. *spot # 459, 465, 466, 467, 468, 469, 470, 471, 472, 473, 474, and 496) and 7 proteins spots (spot # 462, 483, 484, 485, 488, 490 and 499) were identified as subunits of membrane-bound complex F(1)F(0)-ATP synthase (see table S2, Additional file 2). In addition, further analyses using PSORTb v.0.4 (a program for bacterial protein subcellular localisation prediction) [26] indicated that there are several other proteins which could be located in the membrane, for instance: spot # 460 (localisation (L): outer membrane; localisation score (LS): 9.49), spot # 461 (L: outer membrane; LS: 9.52), spot # 463, 464, 487 and 488 (L: outer membrane, LS: 9.49), spot # 478 (L: periplasmic, LS: 4.48), spot # 481 (L: Cytoplasmic membrane; LS: 0.51), spot # 493 (L: Cytoplasmic membrane; LS: 0.51), spot # 494 (L: Cytoplasmic membrane; LS: 0.51), spot # 495 (L: Cytoplasmic membrane; LS: 0.51).

Curiously, when comparing the data here presented with previous reports, it become visible that several proteins here identified are also present in the membrane proteome of other *A. baumannii *strains, including clinical isolates [13-15] (see table S2, Additional file 2). It is therefore tempting to propose that these proteins are constitutively present in the membrane of *A. baumannii *and if that so, then this valuable information can be utilised for development of new drugs, vaccines and/or diagnostic.

A number of Omps associated with the transport across the membrane of several compounds were identified in the present study. Including proteins such as putative copper receptor (OprC, spot # 459), which allows the penetration of small cations, a putative Omp (OprD, spot # 465) that in *Pseudomonas aeruginosa *has been demonstrated to be involved in the uptake of basic amino acids, small peptides and of imipenem [27]; putative glucose-sensitive porin (OprB-like spot # 466), this porin is often referred to as carbohydrate selective porin, since it acts as a central component of glucose, mannitol, glycerol, and fructose transport across the outer membrane [28]; Omp 38 precursor (spot # 467) belonging to the OmpA family, which has been suggested to be involved with the transport of β-lactams and saccharides up to approximately 800 Da [29,30]; an Omp (with homology with Omp 33-36 kDa, spot # 468) involved in the transport of water and carbapenems [31]; Omp CarO (spot # 469 & spot # 472), which is essential for the uptake of L-ornithine and participates in the selective uptake of carbapenems and other basic amino acids in *A. baumannii *[32].

Hence, the results indicate that the most abundant membrane proteins were those involved in the transport of different components (amino acid, sugars, and fatty acids), which suggests that *A. baumannii *have an adaptable metabolism that utilises a wide range of carbon sources.

### Cytoplasmatic extract

MALDI-TOF/TOF analyses of cytoplasmatic extract revealed that of the 155 identified spots, 12 were identified as hypothetical proteins (see table S1, Additional file 1) and of those 9 could be functionally classified according COG (information obtained by MASCOT analyses). The three most representative groups are those related to amino acid and transport metabolism (29), translation, ribosomal structure and biogenesis (28) and energy production and conversion (14). Also there are other important groups (see table S1, Additional file 1 and Fig. 5). Analyses of the cytoplasmatic proteins revealed that much of the *A. baumannii *proteome is dedicated to the metabolism of a wide range of components. One of the most highly represented classes of proteins was that involved in amino acid transport and metabolism. This comprised proteins involved in the metabolism of amino acid such as glutamine (spot # 8), serine (spot # 64 & 67), aspartate (spot # 103), methionine (spot # 104), cysteine (spot # 182 & 186), trypthophan (spot # 233), lysine (spot # 95; 176 & 211), glutamate (spot # 85), proline (spot # 75), threonine (spot # 108), and histidine (spot # 120; 182 & 234). In addition, the multifunctional proteins included proteins such as branched-chain amino acid transferase, which catalyses the transamination of the branched-chain amino acids (spot # 132) leucine, isoleucine and valine to their respective alpha-keto acids [33]. Taking into consideration the number, level of expression (in a population with an average of % spot volume of 0.21 there was: spot # 67 0.6 ± 0.04; spot # 123 0.55 ± 0.03; spot # 285 1.13 ± 0.36; see also Fig. 2) and the diversity of proteins within this group, it is reasonable to conclude that the amino acid metabolism is in fact a major pillar of the overall *A. baumannii *metabolism.

Interestingly, a phospho-2-dehydro-3-heoxyheptonate aldolase (spot # 123) was identified; in *E. coli *this type of synthetase catalyses the first step in aromatic amino acid biosynthesis from chorismate [34]. Earlier studies have demonstrated that species of the genus *Acinetobacter *are capable of degrading aromatic compounds [24,35-37]. The capacity of *Acinetobacter *spp. to degrade aromatic compounds is of particular interest for bioremediation studies [38] and also for the development of new antibiotics [24]. Therefore, the protein here identified and its possible involvement in aromatic compound degradation may be subject of further investigation.

As mentioned above, a low molecular weight protein named nitrogen assimilation regulatory protein P-II 2 (spot # 352) was identified. In *E. coli *this protein regulates the assimilation of nitrogen by modulating the activity of glutamase synthase [39], this regulatory mechanism allows the cell to adjust the glutamase synthase activity quickly - in response to alterations in the abundance of the preferred nitrogen source, ammonia [39]. Although further investigation is required, this suggests that nitrogen is another source of energy utilised by this microorganism.

In concordance with previous genomic studies [8,9,40] that indicated the inability of *A. baumannii *to catabolise glucose, the current proteomic analysis did not detect any enzymes such as hexokinase, glucosekinase or proteins of the phosphotransferase transport system essential for glycolysis. However, it has to be referred the already mentioned presence of the putative glucose porin sensitive outer membrane (OprB-like) suggests the uptake of glucose from *A. baumannii*. Hence, this information supports the existence of a route passing through the Entner-Doudoroff pathway [41], in which the oxidation of glucose in the periplasm leads to pyruvate formation. Moreover, the presence of both fructose-1,6-bisphosphatase (spot # 138) and fructose-1,6-bisphosphate aldolase (spot # 156), shows that the bacterium in question is capable of catabolising fructose. Hence, it would be interesting to investigate further the capability of these and other sugars to support growth of this bacterium.

Identification of the selected alkaline spots revealed that most were ribosomal proteins (see table S1, Additional file 1) as previously described in other microbe studies [42]. In addition, to ribosome-associated proteins, several other proteins of the translational machinery were also detected. For instance, translation elongation factors EF-G (spot # 5), EF-Tu (spot # 100), EF-Ts (spot # 178), EF-P (spot # 235) and ribosome releasing factor (spot # 248). Ribosomes, ribosome-associated proteins and proteins associated with translation are targets for many antibiotics, which interfere with translation through different molecular mechanisms of action [12]. As far as we know, this is the first proteomic study that has identified such a large number of ribosomes and ribosome-associated proteins in the *A. baumannii *proteome, and we believe that the data provided here can serve as preliminary data for further studies on this bacterium, including studies of antibiotic drug action.

### Proteins with potential role in drug resistance, stress response and in virulence

A recent description of the *A. baumannii *ATCC 17978 genome revealed that this strain has acquired several genes from its environment, these foreign genes are clustered in small (>10 kb) regions called putative alien islands (pAs) [9]. In addition, it was reported that ATCC 17978 genome have a total of 28 pAs and some of these are associated with drug resistance, virulence, iron uptake, metabolism and others [9]. Of the identified proteins, only 26 out of 192 were encoded by genes mapped in these pAs (see table S1, Additional file 1 and table S2, Additional file 2). This led us to conclude that most of the expressed proteins correspond to genes located in the native *A. baumannii *chromosome. Hence, it is tempting to speculate that those pAs are mainly expressed under specific conditions, *e.g. *stress.

Although for the most cases it was not clear the association between the protein and its respective pAs function, there were a few examples where it was possible to establish a more direct association. For instance, heat-shock proteins (Hsps) namely chaperone Hsp60 (spot # 35), and Co-chaperonin GroES (spot # 347), are encoded by genes situated in drug resistance pA 23 [9]. Many of the Hsps are chaperones and are important for protection against environmental stress and provide tolerance to high temperature [43,44], nutrient deprivation [45], salinity and osmotic stress [46]. Moreover, taking into account that Hsp60 chaperones were by far one of the most abundant protein spot (spot # 35 1.13 ± 0.8); and considering the presence of other Hsps (spot # 1, 2 and 393), it appears reasonable to suggest that these Hsps play a relevant role in the responses of *A. baumannii *to environmental changes and in the survival and adaptation to stress conditions. At this respect, a recent report have shown downregulation of Hsp60 proteins associated with resistance to colistin concomitantly to a decrease in bacterial fitness in an *A. baumannii *isolate [47]. In addition to Hsps, the Universal stress proteins (Usps) here identified (spot # 332, 333 and 498) are encoded by genes located in pA 18, which is associated with drug resistance [9]. In *E. coli *the expression level of these small cytoplasmatic proteins increase when cells are exposed to stress including CdCl_2_, H_2_O_2_, DNP, CCCP and osmotic stress [48], moreover Usp enhances the rate of cell survival during prolonged exposure to stress conditions [48]. In *A. baumannii*, the function of Usps remain elusive, recently we have observed that the expression of these proteins increase at late phases of *in vitro *growth (data not published), therefore Usps can be an attractive target for further studies concerning drug resistance.

Within the stress response context it is worthy to refer the presence of proteins known to play a relevant role in response to oxidative stress such as alkyl hydroperoxide reductase (spot # 271 and 273), superoxide dismutase (spot # 247), and glutathione peroxidase (478).

Here we have identified several proteins encoded by genes situated in pAs with a predicted role in virulence, however again it is not straightforward the association of those proteins with virulence. Nevertheless, amongst the proteins identified in this report, a few may be more directly related to virulence. For example, a protein blast alignment (data not shown) of the here identified OmpW revealed that this protein shares a high homology with putative virulence-related OmpW (gi|188591914) from *Cupriavidus taiwanensis*. Also, it has been suggested that Omp 38 (spot # 467) may act as a potential virulence factor inducing apoptosis of epithelial cells in the early stage of *A. baumannii *infection [[49], Choi, 2008 #392]. In addition, a recently publication describing the proteome of OMVs of a clinical *A. baumannii *presented evidence that virulence-associated proteins such as Omp38 (gi|126642864), OmpW (gi|126640380) and bacterioferritin (gi|126640856) are transported in OMVs, where they have been suggested to play an important role during *in vivo *infection [15]. It is of interest that whereas Kwon *et al. *(2009) [15] identified proteins such as 30S ribosomal protein S1 (spot # 3), Glutamine synthetase (spot # 8), Malic enzyme (spot # 21), Chaperone Hsp60 (spot # 35) and others (see table S1, Additional file 1) associated to the OMVs, we find these proteins exclusively in the cytoplasmatic fraction. In the referred study, the authors do not rule out the possibility of the entrapment of these proteins by unknown mechanism. If this is the case, then the data gathered here, together with that presented in the referred study provide a list of potential candidate cytoplasmatic proteins that can be transported in OMVs.

Furthermore, in the current study a putative type III effector (spot # 353) was identified, and the interactions between *Pseudomonas synringae*-host interaction is mediated in great part by effector proteins, which are injected into plant cells by type III secretion [50]. In *A. baumannii *the function of type III effector has yet to be elucidated, although it probably participates in bacterium-host interactions.

The specific virulence factors or pathogenic mechanisms of this bacterium remain elusive [51]. In light of the present results, it would be interesting to use a proteomic approach to investigate the expression of these and other proteins with a potential role in virulence, in the presence of virulence-induced stimuli or in the process of host-bacteria interactions (experiments are currently in progress).

## Conclusion

Here we report for the first time a 2-DE based analysis, of both membrane and cytoplasmatic fraction of the opportunistic pathogen *A. baumannii*. Analysis of the most abundant proteins of this microorganism indicates that *A. baumannii *has a versatile and robust metabolism capable of utilising a wide range of nutrient sources. In addition, this study revealed the presence of highly expressed proteins such as porins (membrane fraction) and chaperones or heat-shock proteins and others, which are likely to play a crucial role not only in mechanisms of virulence and drug resistance, but also in adaptive environmental responses, which are probably related to the persistence in hospital settings, one of the hallmark of this microorganism. Thus, this proteomic approach provides us with new molecular tools to investigate the complexity of mechanisms operating during virulence and adaptive responses of this pathogen, which has been recently outlined by the Infectious Diseases Society of America as one of six important highly dangerous drug resistant microbes in hospitals worldwide.

## Abbreviations

2-DE: two-dimensional gel electrophoresis; Hsp: heat-shock protein; MALDI: matrix-assisted laser desorption ionisation; Omp: outer membrane protein; pAs: putative alien islands; TOF: time of flight.

## Competing interests

The authors declare that they have no competing interests.

## Authors' contributions

NCS, MPC and GB have made substantial contributions to conception and design of the experiments. NCS and MPC carried out the 2-DE gels experiments and performed the MALDI-TOF/TOF acquisition and interpretation of data. NCS and JRP carried out the Image Master data analysis and interpretation. MPC, CG and MJB were responsible for cell culture and protein sample. NCS, CPC and GB have been involved in drafting the manuscript or revising it critically for important content. All authors read and approved the final manuscript.

## Supplementary Material

Additional file 1 **Table S1 - MALDI-TOF/TOF identification of *Acinetobacter baumannii *cytoplasmatic protein spots**. Identified proteins are listed with 2-DE spot numbers, protein description, theoretical Mr and p*I*, in gel Mr and *pI*, accession numbers, functional class, values resulting from Mascot data (score, number of matched peptides and percentage coverage) and information concerning the respective pAs. Click here for file

Additional file 2 **Table S2 - MALDI-TOF/TOF identification of *Acinetobacter baumannii *membrane protein spots**. Identified proteins are listed with 2-DE spot numbers, protein description, theoretical Mr and p*I*, in gel Mr and *pI*, accession numbers, functional class, values resulting from Mascot data (score, number of matched peptides and percentage coverage) and information concerning the respective pAs. Click here for file

Additional file 3 **Table S3 - MALDI-TOF/TOF identification of *Acinetobacter baumannii *proteins with the respective matched peptide sequences**. Complementary results of the MALDI-TOF/TOF and MASCOT analyses. For each protein the number and the sequences of matched peptides and corresponding NCBI identifier are provided. Click here for file

## References

[B1] Charnot-Katsikas A, Dorafshar AH, Aycock JK, David MZ, Weber SG, Frank KM (2009). Two Cases of Necrotizing Fasciitis Due to *Acinetobacter baumannii*. J Clinical Microbiol.

[B2] Fournier P, Richet H (2006). The epidemiology and control of *Acinetobacter baumannii *in health care facilities. Clin Infect Dis Dis.

[B3] Katsaragakis S, Markogiannakis H, Toutouzas K, Drimousis P, Larentzakis A, Theodoraki E-M, Theodorou D (2008). *Acinetobacter baumannii *infections in a surgical intensive care unit: predictors of multi-drug resistance. World J Surg.

[B4] Bou G, Cervero G, Dominguez MA, Quereda C, Martinez-Beltran J (2000). Characterization of a nosocomial outbreak caused by a multiresistant *Acinetobacter baumannii *strain with a carbapenem-hydrolyzing enzyme: high-level carbapenem resistance in *A. baumannii *is not due solely to the presence of beta-lactamases. J Clin Microbiol.

[B5] Jones M, Draghi D, Thornsberry C, Karlowsky J, Sahm D, Wenzel R (2004). Emerging resistance among bacterial pathogens in the intensive care unit - a European and North American Surveillance study (2000-2002). Ann Clin Microbiol Antimicrob.

[B6] Vila J, Marti S, Sanchez-Cespedes J (2007). Porins, efflux pumps and multidrug resistance in *Acinetobacter baumannii*. J Antimicrob Chemother.

[B7] Fournier P-E, Vallenet D, Barbe V, Audic S, Ogata H, Poirel L, Richet H, Robert C, Mangenot S, Abergel C (2006). Comparative genomics of multidrug resistance in *Acinetobacter baumannii*. PLoS Genet.

[B8] Vallenet D, Nordmann P, Barbe V, Poirel L, Mangenot S, Bataille E, Dossat C, Gas S, Kreimeyer A, Lenoble P (2008). Comparative analysis of *Acinetobacters*: three genomes for three lifestyles. PLoS ONE.

[B9] Smith MG, Gianoulis TA, Pukatzki S, Mekalanos JJ, Ornston LN, Gerstein M, Snyder M (2007). New insights into *Acinetobacter baumannii *pathogenesis revealed by high-density pyrosequencing and transposon mutagenesis. Genes Dev.

[B10] Keller M, Hettich R (2009). Environmental proteomics: a paradigm shift in characterizing microbial activities at the molecular level. Microbiol Mol Biol Rev.

[B11] Gupta N, Tanner S, Jaitly N, Adkins JN, Lipton M, Edwards R, Romine M, Osterman A, Bafna V, Smith RD, Pevzner PA (2007). Whole proteome analysis of post-translational modifications: applications of mass-spectrometry for proteogenomic annotation. Genome Res.

[B12] Brötz-Oesterhelt H, Bandow JE, Labischinski H (2005). Bacterial proteomics and its role in antibacterial drug discovery. Mass Spectrom.

[B13] Martí S, Sánchez-Céspedes J, Oliveira E, Bellido D, Giralt E, Vila J (2006). Proteomic analysis of a fraction enriched in cell envelope proteins of *Acinetobacter baumannii*. Proteomics.

[B14] Siroy A, Cosette P, Seyer D, Lemaitre-Guillier C, Vallenet D, Van Dorsselaer A, Boyer-Mariotte S, Jouenne T, De E (2006). Global comparison of the membrane subproteomes between a multidrug-resistant *Acinetobacter baumannii *strain and a reference strain. J Proteome Res.

[B15] Kwon S-O, Gho YS, Lee JC, Kim SI (2009). Proteome analysis of outer membrane vesicles from clinical *Acinetobacter baumannii *isolate. FEMS Microbiol Lett.

[B16] Molloy MP, Herbert BR, Slade MB, Thierry R, Nouwens AS, Williams KL, Gooley AA (2000). Proteomics analysis of the *Escherichia coli *outer membrane. Eur J Biochem.

[B17] Bradford M (1976). A rapid and sensitive method for the quantification of microgram quantities of protein utilizing the principle of protein-dye binding. Anal Biochem.

[B18] Ramagli L (1999). Quantifying protein in 2-D PAGE solubilisation buffers. Methods Mol Biol.

[B19] Laemmli UK (1970). Cleavage of structural proteins during the assembly of head proteins of bacteriophage T4. Nature.

[B20] Blum H, Beier H, Gross H (1987). Improved silver staining of plant proteins, RNA and DNA in polyacrylamide gels. Electrophoresis.

[B21] Pandey A, Andersen J, Mann M (2000). Use of mass spectrometry to study signalling pathways. Sci STKE.

[B22] Sechi S, Chait BT (1998). Modification of cysteine residues by alkylation. A tool in peptide mapping and protein identification. Anal Chem.

[B23] Lamberti C, Pessione E, Giuffrida MG, Mazzoli R, Barello C, Conti A, Giunta C (2007). Combined cup loading, bis(2-hydroxyethyl) disulfide, and protein precipitation protocols to improve the alkaline proteome of *Lactobacillus hilgardii*. Electrophoresis.

[B24] Park S-H, Kim J-W, Yun S-H, Leem S-H, Kahng H-Y, Kim SI (2006). Characterization of beta-ketoadipate pathway from multi-drug resistance bacterium, *Acinetobacter baumannii *DU202 by proteomic approach. J Microbiol.

[B25] Tatusov RL, Koonin EV, Lipman DJ (1997). A genomic perspective on protein families. Science.

[B26] Gardy JL, Laird MR, Chen F, Rey S, Walsh CJ, Ester M, Brinkman FS (2005). PSORTb v.2.0: Expanded prediction of bacterial protein subcellular localization and insights gained from comparative proteome analysis. Bioinformatics.

[B27] Nikaido H (2003). Molecular basis of bacterial outer membrane permeability revisited. Microbiol Mol Biol Rev.

[B28] Wylie JL, Worobec EA (1995). The OprB porin plays a central role in carbohydrate uptake in *Pseudomonas aeruginosa*. J Bacteriol.

[B29] Gribun A, Nitzan Y, Pechatnikov I, Hershkovits G, Katcoff DJ (2003). Molecular and structural characterization of the HMP-AB gene encoding a pore-forming protein from a clinical isolate of *Acinetobacter baumannii*. Curr Microbiol.

[B30] Nitzan Y, Pechatnikov I, Bar-El D, Wexler H (1999). Isolation and characterization of heat-modifiable proteins from the outer membrane of *Porphyromonas asaccharolytica *and *Acinetobacter baumannii*. Anaerobe.

[B31] del Mar Tomas M, Beceiro A, Perez A, Velasco D, Moure R, Villanueva R, Martinez-Beltran J, Bou G (2005). Cloning and functional analysis of the gene encoding the 33- to 36-kilodalton outer membrane protein associated with carbapenem resistance in *Acinetobacter baumannii*. Antimicrob Agents.

[B32] Mussi MA, Relling VM, Limansky AS, Viale AM (2007). CarO, an *Acinetobacter baumannii *outer membrane protein involved in carbapenem resistance, is essential for L-ornithine uptake. FEBS Let.

[B33] Harper AE, Miller RH, Block KP (1984). Branched-Chain Amino Acid Metabolism. Annual Review of Nutrition.

[B34] Shumilin I, Kretsinger R, Bauerle R (1999). Crystal structure of phenylalanine-regulated 3-deoxy-D-arabino-heptulosonate-7-phophate synthase from Escherichia coli. Structure.

[B35] Giuffrida MG, Pessione E, Mazzoli R, Dellavalle G, Barello C, Conti A, Giunta C (2001). Media containing aromatic compounds induce peculiar proteins in *Acinetobacter radioresistens*, as revealed by proteome analysis. Proteomics.

[B36] Pessione E, Giuffrida MG, Barello C, Mazzoli R, Fortunato D, Conti A, Giunta C (2003). Membrane proteome of *Acinetobacter radioresistens *S13 during aromatic exposure. Proteomics.

[B37] Kim E-A, Kim JY, Kim S-J, Park KR, Chung H-J, Leem S-H, Kim SI (2004). Proteomic analysis of *Acinetobacter lwoffii *K24 by 2-D gel electrophoresis and electrospray ionization quadrupole-time of flight mass spectrometry. J Microbio Methods.

[B38] Kim SI, Choi J-S, Kahng H-Y (2007). A proteomics strategy for the analysis of bacterial biodegradation pathways. OMICS.

[B39] Jiang P, Zucker P, Atkinson MR, Kamberov ES, Tirasophon W, Chandran P, Schefke BR, Ninfa AJ (1997). Structure/function analysis of the PII signal transduction protein of *Escherichia coli*: genetic separation of interactions with protein receptors. J Bacteriol.

[B40] Iacono M, Villa L, Fortini D, Bordoni R, Imperi F, Bonnal RJ, Sicheritz-Ponten T, De Bellis G, Visca P, Cassone A, Carattoli A (2008). Whole genome pyrosequencing of an epidemic multidrug resistant *Acinetobacter baumannii *strain belonging to the European clone II group. Antimicrob Agents Chemother.

[B41] Taylor WH, Juni E (1961). Pathways for biosynthesis of a bacterial capsular polysaccharide II.: Carbohydrate metabolism and terminal oxidation mechanisms of a capsule-producing Coccus. J Bacteriol.

[B42] Kolker E, Purvine S, Galperin MY, Stolyar S, Goodlett DR, Nesvizhskii AI, Keller A, Xie T, Eng JK, Yi E (2003). Initial proteome analysis of model microorganism *Haemophilus influenzae *strain Rd KW20. J Bacteriol.

[B43] Lu Q, Han J, Zhou L, Coker JA, DasSarma P, DasSarma S, Xiang H (2008). Dissection of the regulatory mechanism of a heat-shock responsive promoter in *Haloarchaea*. Nucleic Acids Res.

[B44] Plesofsky-Vig N, Brambl R (1985). Heat shock response of *Neurospora crassa*: protein synthesis and induced thermotolerance. J Bacteriol.

[B45] Spence J, Cegielska A, Georgopoulos C (1990). Role of *Escherichia coli *heat shock proteins DnaK and HtpG (C62.5) in response to nutritional deprivation. J Bacteriol.

[B46] Bhagwat AA, Apte SK (1989). Comparative analysis of proteins induced by heat shock, salinity, and osmotic stress in the nitrogen-fixing cyanobacterium *Anabaena *Csp. strain L-31. J Bacteriol.

[B47] Fernández-Reyes M, Rodríguez-Falcón M, Chiva C, Pachón J, Andreu D, Rivas L (2009). The cost of resistance to colistin in *Acinetobacter baumannii*: a proteomic perspective. Proteomics.

[B48] Nystrom T, Neidhardt FC (1994). Expression and role of the universal stress protein, UspA, of *Eschrichia coli *during growth arrest. Mol Microbiol.

[B49] Choi CH, Lee EY, Lee YC, Park TI, Kim HJ, Hyun SH, Kim SA, Lee S-K, Lee JC (2005). Outer membrane protein 38 of *Acinetobacter baumannii *localizes to the mitochondria and induces apoptosis of epithelial cells. Cell Microbiol.

[B50] Lindeberg M, Stavrinides J, Chang JH, Alfano JR, Collmer A, Dangl JL, Greenberg JT, Mansfield JW, Guttman DS (2005). Proposed guidelines for a unified nomenclature and phylogenetic analysis of type III Hop effector proteins in the plant pathogen *Pseudomonas syringae*. Mol Plant Microbe Interact.

[B51] Choi CH, Hyun SH, Lee JY, Lee JS, Lee YS, Kim SA, Chae J-P, Yoo SM, Lee JC (2008). *Acinetobacter baumannii *outer membrane protein A targets the nucleus and induces cytotoxicity. Cell Microbiol.

