# Supplementary material for: 2-DE analysis indicates that Acinetobacter baumannii displays a robust and versatile metabolism
Source: Proteome Sci. 2009 Sep 28;7:37. doi: 10.1186/1477-5956-7-37 (PMC2761859; doi:10.1186/1477-5956-7-37)
Supplement: Additional file 1 — Table S1 - MALDI-TOF/TOF identification of Acinetobacter baumannii cytoplasmatic protein spots. Identified proteins are listed with 2-DE spot numbers, protein description, theoretical Mr and pI, in gel Mr and pI, accession numbers, functional class, values resulting from Mascot data (score, number of matched peptides and percentage coverage) and information concerning the respective pAs. [file 1477-5956-7-37-S1.PDF]

Table S1. MALDI-TOF/TOF Identification of *Acinetobacter baumannii* cytoplasmatic protein spots. The databases were queried with resulting Mascot data files.

Spot no.	Identified protein	Theoretical kDa/p*I*	In gel kDa/p*I*	Acession no.	Class [a)]	Score	Match pept.[b)]	% Cov.[c)]	pA no. [d)]
1	Chaperone Hsp 70	64.4/4.74	58/4	gi|126642981[*]	PTC	530	8	16	24
2	Chaperone Hsp 90	71.8/4.93	58/5	gi|169634585	PTC	216	3	7	-
3	30S ribosomal protein S1	52.6/5.57	57/5	gi|126641617[***]	TRB	167	3	7	11
5	Elongation factor G	73.8/4.9	60 /5	gi|126640918[***]	TRB	488	7	12	-
6	Polynucleotide phosphorylase/polyadenylase	71.2/4.97	59/5	gi|126640439[*]	TRB	260	4	7	-
8	Glutamine synthetase	29/5.11	57/5	gi|126642389[**]	ATM	132	3	11	21
9	Phosphoenolpyruvate carboxykinase	62.5/5.27	56/5	gi|126642702	EPC	173	3	6	-
21	Malic enzyme	73.6/5.4	59/5	gi|126642378[**]	EPC	272	5	7	-
33	Trigger factor spetum formation molecular chaperone	39.8/4.61	51/4	gi|126640548	PTC	313	4	18	-
34	Chaperone Hsp60	50.0/4.71	48 /4	gi|126642698[***]	PTC	510	6	22	23
35	Chaperone Hsp60	50/.04.71	52/5	gi|126642698[***]	PTC	585	5	21	23
36	Chaperone Hsp60	50.0/4.71	54/5	gi|126642698[***]	PTC	135	1	3	23
38	Phosphoglycerate mutase III cofactor independent	52.2/5.2	54/5	gi|126640323[*]	CTM	482	8	21	-
40	Phosphoglycerate mutase III cofactor independent	52.2/5.2	54/5	gi|126640323[*]	CTM	89	4	2	-
42	NAD-linked malate dehydrogenase, Rossman fold	63.0/5.32	54/5	gi|169797658	EPC	104	2	3	-
46	Urocanate hydratase	61.6/5.64	54 /5	gi|169634859[*]	ATM	106	5	11	-
47	Urocanate hydratase	61.6/5.64	54/6	gi|169634859[*]	ATM	513	8	16	-
48	Hypothetical protein A1S_2187	25.5/5.97	51/5	gi|126642232	NTM	167	2	10	22

Table S1. continued

Spot no.	Identified protein	Theoretical kDa/p*I*	In gel kDa/p*I*	Acession no.	Class [a)]	Score	Match pept.[b)]	% Cov.[c)]	pA no. [d)]
54	F0F1 ATP synthase β-subunit	50.3/5.03	46/5	gi|162286755**	EPC	213	4	15	-
58	F0F1 ATP synthase α-subunit	55.5/5.29	49/5	gi|162286757*	Mult.	359	5	11	-
59	F0F1 ATP synthase α-subunit	55.5/5.29	48/5	gi|162286757*	Mult.	458	10	24	-
64	Serine hydroymethyltranferase	40.5/6.22	46/5	gi|126642348	ATM	105	3	11	20
66	Putative protein (DcaP-like)	44.8/5.79	47/5	gi|126642784	CEOM	80	2	7	-
67	Serine hydroymethyltranferase	40.5/6.22	45/5	gi|126642348	ATM	168	3	11	20
75	Gamma-glutamyl phosphate reductase	39.8/5.81	43/6	gi|126640560	ATM	133	3	12	-
80	Dihydrolipoamide dehydrogenase	42.5/5.64	46/6	gi|126642748**	RR	229	4	17	-
85	Diaminobutyrate-2-oxoglutarate aminotransferase	46.7/6.1	43/6	gi|126642490*	ATM	110	2	5	-
88	Cell division protein FtsZ	38.5/4.96	42/5	gi|126643338	CDCP	107	2	12	-
92	Enolase	43.1/5.12	41/5	gi|126641943	CTM	193	3	10	-
94	Dihdrolipoamide succinyltranferase component of 2-oxoglutarate dehydrogenase complex (E2)	39.9/5.14	45/5	gi|126642747**	EPC	210	4	15	-
95	Dihdrolipoamide succinyltranferase component of 2-oxoglutarate dehydrogenase complex (E2)	39.9/5.14	44/5	gi|126642747**	EPC	243	5	17	-
96	Histidinol dehydrogenase	38.9/5.09	41/5	gi|126640749	ATM	129	3	10	4
99	Beta-ketoacyl-ACP synthase I (3-oxoacyl-[acyl-carrier-protein] synthase I)	43.4/5.20	42/5	gi|169634061	Mult.	356	6	12	-
100	Elongation factor Tu	43.1/5.21	42/5	gi|162286753	TRB	172	3	10	-
103	Aspartate aminotransferase A	40..9/5.1	42./5	gi|126642544*	ATM	351	4	13	-

Spot no.	Identified protein	Theoretical kDa/p*I*	In gel kDa/p*I*	Acession no.	Class [a)]	Score	Match pept.[b)]	% Cov.[c)]	pA no. [d)]
104	Methionine adenosyltranferase	31..9/6.4	40/5	gi|126641564[*]	ATM	184	4	16	10
105	Hypothetical protein A1S_2277	33.6/5.65	39./5	gi|126642318	ATM	303	4	16	20
108	Threonine synthase pyridoxal-5´-phosphate-dependent enzyme	32.9/5.02	38/5	gi|126640331	ATM	477	7	32	-
110	Succinylornithine transaminase	29.6/5.4	44/5	gi|126643147[*]	ATM	61	1	6	-
114	Hypothetical protein A1S_3388	38.8/5.61	39/6	gi|126643395	CoE	76	2	7	-
115	WecE protein	32.3/5.91	36/6	gi|126640168[*]	ATM	104	2	9	20
120	Imidazolonepropionase	34.6/5.64	38/6	gi|126643410	ATM	195	3	11	-
123	Phospho-2-dehydro-3-heoxyheptonate aldolase	41.8/5.85	36/6	gi|126641709	ATM	77	2	7	-
124	D-3-phophoglycerate dehydrogenase	33.8/5.86	39/6	gi|126643167	ATM	135	3	10	-
132	Branched-chain amino acid transferase	30.6/6.31	31/6	gi|126642928	Mult.	102	2	9	-
134	Succinyl-CoA synthetase beta chain	36.9/4.9	39/5	gi|126642749	CoE	95	1	4	-
135	Phosphoglycerate kinase	35.5/4.47	37/4	gi|126641588	EPC	81	2	10	-
137	Succinyl-CoA synthetase α-chain	25.0/5.07	36/5	gi|126642750[**]	CoE	64	2	14	-
138	Fructose-1,6-bisphosphatase	28.8/4.9	35/4	gi|126642631	CTM	61	2	8	-
140	Outer membrane protein (Omp)38 precursor	38.4/5.32	37/5	gi|126642864[*]	CEOM	364	6	17	-
145	DNA-directed RNA polymerase subunit alpha	37.3/5.1	39/5	gi|158513671	RPS	379	8	27	-
149	Omp 38 precursor	38.4/5.32	36/5	gi|126642864[*]	CEOM	256	4	11	-
154	Succinyl-CoA synthetase α-chain	25.0/5.07	31/5	gi|126642750[**]	CoE	78	9	44	-
156	Fructose-1,6-bisphosphate aldolase, class II	37.5/5.43	33/6	gi|162286736	CTM	212	6	30	-

Table S1.continued

Spot no.	Identified protein	Theoretical kDa/p*I*	In gel kDa/p*I*	Acession no.	Class [a)]	Score	Match pept.[b)]	% Cov.[c)]	pA no. [d)]
158	Succinyl-CoA synthetase α-chain	25.0/5.07	30/6	gi|126642750**	CoE	128	2	14	-
171	Thioredoxin reductase	34.0/4.96	29/5	gi|193076628	RR	174	2	11	-
172	Putative flavohemoprotein	25.3/4.68	29/5	gi|126643100	EPC	56	1	7	-
173	Malonyl-CoA-[acyl-carrier-protein] transacylase	34.6/5.12	29/5	gi|169634114	CoE	75	1	3	-
174	Putative intercellular/amidase	32.1/5.11	36/5	gi|193078218	ATM	192	4	18	-
176	Dihydrodipicolinate synthase	25.9/5.31	47/5	gi|126643430	ATM	192	6	33	-
178	Elongation factor Ts	30.8/5.27	30/5	gi|126642362*	TRB	794	10	33	-
180	Malate dehydrogenase	35.3/5.2	30/5	gi|152032571	RR	443	10	40	-
182	Subunit of cysteine synthase A and O-acetylserine sulfhydrolase A, PLP-dependent enzyme	35.2/5.23	31/5	gi|169632185	ATM	72	2	7	-
186	Cysteine synthase B	26.8/5.26	29/5	gi|126640646	ATM	98	3	20	-
191	NADH-dependent enoyl-ACP reductase	28.8/5.61	27/6	gi|126640605*	RR	76	3	18	-
192	NADH-dependent enoyl-ACP reductase	28.8/5.61	28/6	gi|126640605*	RR	115	3	14	-
193	Hypothetical protein A1S_1833	31.0/5.74	28/6	gi|193077447	GF	201	3	15	-
194	Putative acetyl-CoA carboxylase, β-subunit	25.0/6.22	28/6	gi|126642893	CoE	101	2	8	-
195	NADH-dependent enoyl-ACP reductase	28.8/5.61	28/6	gi|126640605*	RR	472	9	30	-
200	50S ribosomal protein L1	21.5/9.10	26/7	gi|126640372**	TRB	327	4	29	-
202	Putative outer membrane protein	24.8/4.70	28/4	gi|126643304**	CEOM	91	3	24	-
204	Conserved hypothetical protein	26.6/4.70	28/5	gi|169797441	U	64	2	11	-

Table S1. continued

Spot no.	Identified protein	Theoretical kDa/p*I*	In gel kDa/p*I*	Acession no.	Class [a)]	Score	Match pept.[b)]	% Cov.[c)]	pA no. [d)]
206	Electron transfer flavoprotein α-subunit	31.5/4.90	28/5	gi|169632682	EPC	142	3	21	-
207	Electron transfer flavoprotein α -subunit	20.7/4.7	28/5	gi|126642662	EPC	338	6	61	-
208	Response regulator (activator) in two-component regulatory (OmpR family)	20.4/5.34	27/5	gi|126640806	S	146	3	26	4
211	2,3,4,5-tetrahydropyridine-2-carboxylate N-succinyltransferase	29.7/5.17	27/5	gi|126642574*	ATM	89	2	10	-
212	Adenylate Kinase	20.6/4.83	27/5	gi|126641073	EPC	293	5	47	-
214	2,3,4,5-tetrahydropyridine-2-carboxylate N-succinyltrans.	29.8/5.17	29/5	gi|126642443	ATM	201	4	19	-
222	Acetyl-coenzyme A carboxylase carboxyl transferase	24.2/5.65	27/ 6	gi|126640675	CoE	201	2	12	-
229	30S ribosomal protein S2	27.6/8.98	27/4	gi|169632956	TRB	128	2	9	-
233	Tryptophan synthase α-chain	28.4/4.84	25/5	gi|162286721	ATM	132	3	20	-
234	1-(5-phophoribosyl)-5-[(5-phosphoribosylamino)methylideamino] imidazole-4-carboxamide isomerase	26.2/4.96	25/5	gi|126643246*	ATM	248	4	20	-
235	Elongation factor P	19.3/4.78	26/5	gi|126642455*	TRB	298	4	25	-
236	Acetoacetyl-CoA transferase alpha subunit	17.7/4.89	28/5	gi|126641777	CoE	242	7	47	-
242	Acetyl-coenzyme A carboxylase carboxyl transferase	24.2/5.65	24/5	gi|126640675	CoE	120	1	5	-
244	Cytidylate Kinase	21.2/5.18	25/6	gi|126641616	EPC	90	2	12	11

Table S1. continued

Spot no.	Identified protein	Theoretical kDa/p*I*	In gel kDa/p*I*	Acession no.	Class [a)]	Score	Match pept.[b)]	% Cov.[c)]	pA no. [d)]
247	Superoxide dismutase	24.2/5.88	24/6	gi|126642383	RR	138	2	9	-
248	Ribosome releasing factor	16.1/5.06	23/6	gi|126642019	TRB	61	1	6	17
251	Putative protease	21.2/5.61	26/6	gi|193078237	ATM	218	3	16	-
254	Putative oxidoreductase	21.9/5.61	25/6	gi|126641974	RR	245	3	20	-
257	Hypothetical protein A1S_0484	20.1/5.77	22/6	gi|126640557	CTM	246	3	23	-
259	Xanthine phosphoribosyltransferase	17.0/6.74	22/6	gi|126643050	ATM	88	3	22	-
265	Omp CarO precursor	24.4/4.7	24/5	gi|126642573[**]	CEOM	80	2	13	-
267	Hps 24 nucleotide exchange factor	21.7/4.86	22/5	gi|126642980	PTC	118	3	17	24
268	Inorganic pyrophosphatase	14.3/5.10	22/5	gi|126640295	CTM	181	3	23	-
269	50S ribosomal protein L4	21.6/9.74	24/5	gi|126643094	TRB	70	2	12	-
270	50S ribosomal protein L4	21.6/9.74	24/5	gi|126643094	TRB	138	4	30	-
271	Alkyl hydroperoxide reductase C22 subunit	18.3/4.96	23/5	gi|126641253	RR	127	2	16	-
273	Alkyl hydroperoxide reductase C22 subunit	18.3/4.96	23/5	gi|126641253	RR	197	3	26	-
283	ATP-dependent Clp protease proteolytic subunit	17.3/5.77	22/6	gi|126640549	PTC	122	5	36	-
285	Peptidyl-prolyl cis-trans isomerase precursor	12.6/5.33	20/5	gi|126642154	ATM	103	2	33	-
296	Transcription elongation factor	17.8/4.88	19/5	gi|126642719	Trans.	152	5	38	23
297	Omp W	20/5.09	22 /5	gi|126640380[**]	CEOM	349	6	44	-
298	Putative peptidoglycan-binding LysM	11.9/4.73	21/5	gi|126640876	S	189	5	59	-
299	Omp W	20/5.09	22 /5	gi|126640380[**]	CEOM	97	2	15	-

Table S1. continued									
Spot no.	Identified protein	Theoretical kDa/p*I*	In gel kDa/p*I*	Acession no.	Class [a)]	Score	Match pept.[b)]	% Cov.[c)]	pA no. [d)]
300	2,3,4,5-tetrahydropyridine-2-carboxylate N-succinyltransferase	29.7/5.17	20/5	gi|126642574	CoE	195	4	20	-
305	Hypothetical protein A1S_0606	11.9/4.44	18/4	gi|126640673	RR	82	1	12	-
307	Hypothetical protein A1S_2843	16.8/4.65	<15/4	gi|193078285	D	247	5	33	-
310	Biotin carboxyl carrier protein of acetyl-CoA carboxylase (BCCP)	13.2/4.81	<15/5	gi|126642055	Mult.	108	2	29	-
314	Nucleoside diphosphate kinase	15.5/5.52	<15/6	gi|169634408	NTM	619	7	55	-
316	50S ribosomal protein	11.8/5.09	<15/6	gi|126640373	TRB	93	5	70	-
317	Nucleoside diphosphate kinase	11.3/4.81	<15/6	gi|126640569	NTM	98	2	35	-
318	50S ribosomal protein L9	15.7/5.67	<15/6	gi|126642218	TRB	349	5	35	-
324	Putative histidine triad family protein	14.1/5.78	<15/6	gi|169794251	S	80	2	18	-
328	Hypothetical protein A1S_0323	9.3/4.53	<15/5	gi|126640405	U	122	3	27	-
331	Putative DNA binding protein	12.4/5.37	<15/6	gi|126640361	GF	132	2	11	-
332	Putative universal stress protein	15.8/5.62	<15/6	gi|193077665	D	410	5	57	18
333	Putative universal stress protein	15.8/5.62	<15/6	gi|193077665	D	443	6	58	18
335	30S ribosomal protein S6	12.7/5.84	<15/6	gi|126642216	TRB	104	2	24	-
336	Hypothetical protein A1S_1624	13.8/6.03	<15/6	gi|126641669	Mult.	137	4	34	-
342	50S ribosomal protein	12.7/4.60	<15/4	gi|126640374	TRB	140	3	27	-
347	Co-chaperonin GroES	10.1/5.09	<15/5	gi|126642699	PTC	56	2	44	23
352	Nitrogen assimilation regulatory protein P-II 2	10.5/5.11	<15/6	gi|126640311	ATM	134	2	20	3
353	Putative type III effector	12/5.41	<15/6	gi|126640467	CEOM	213	3	45	-
360	Putative toluene tolerance protein Ttg2F	9.2/5.74	<15/6	gi|126640746	Trans	61	1	12	4
361	Chaperone Hsp60	50/4.71	59/6	gi|126642698[***]	PTC	134	2	8	-

Table S1. continued

Spot no.	Identified protein	Theoretical kDa/p*I*	In gel kDa/p*I*	Acession no.	Class [a)]	Score	Match pept.[b)]	% Cov.[c)]	pA no. [d)]
362	Dihydrolipoamide dehydrogenase (Glycine oxidation Lfactor)	51.3/5.81	60/6	gi|169632626	RR	547	5	19	-
364	Transcription termination factor Rho	47.5/7.02	56/6	gi|169634328	Trans	347	10	15	-
370	Hypothetical protein A1S_2371	42.5/8.66	55/8	gi|126642408	Mult.	92	3	7	-
373	Putative short-chain dehydrogenase	48.5/8.59	55/9	gi|193077656	RR	139	5	20	18
379	Transcription termination factor Rho	47.4/7.02	54/7	gi|169634328	Trans	140	3	10	-
385	Mutarotase precursor	37.1/8.49	52/8	gi|126641015	CTM	96	2	12	-
389	Hypothetical protein A1S_3473	43.8/8.94	52/9	gi|126640111	Mult.	160	3	5	-
393	Heat shock protein Hsp40	37.6/6.15	54/7	gi|126643450	PTC	208	6	23	-
394	Lipoyl synthase	35.7/6.57	52/7	gi|162286727	CoE	67	2	8	-
400	Hypothetical protein A1S_0015	53.3/6.08	30/6	gi|126640130	U	175	3	12	-
404	Electron transfer flavoprotein β-subunit	26.5/6.48	29/6	gi|169632681	EPC	309	7	44	-
405	Putative hydrolase	30.7/8.62	31/8	gi|126642696	GF	132	1	5	23
406	30S ribosomal protein S2	11.2/4.3	30/9	gi|126642363	TRB	60	1	28	-
407	50S ribosomal protein L1	23.8/9.54	29/9	gi|169634597	TRB	504	7	53	-
408	Electron transfer flavoprotein β-subunit	23.1/5.2	29/6	gi|126642663*	EPC	625	7	51	-
410	FKBP-type peptidyl-prolyl cis-trans isomerase	26.4/6.44	29/7	gi|126640161	PTC	77	3	9	-
412	30S ribosomal protein S4	23.3/10.1	29/7	gi|169632374	TBR	277	8	34	-
418	50S ribosomal protein L3	22.8/9.87	28/10	gi|126643095	TRB	141	2	13	-
427	Putative signal peptide	21.0/9.32	24/9	gi|193077163	S	238	4	31	-
428	50S ribosomal protein L3	22.8/9.87	25/10	gi|126643095	TRB	491	7	41	-

Table S1. continued

Spot no.	Identified protein	Theoretical kDa/p*I*	In gel kDa/p*I*	Acession no.	Class [a)]	Score	Match pept.[b)]	% Cov.[c)]	pA no. [d)]
432	50S ribosomal protein L6	19.1/9.75	25/10	gi|169632365	TRB	306	5	28	-
437	50S ribosomal protein L5	17.1/9.67	20/10	gi|126643084**	TRB	156	3	32	-
439	50S ribosomal protein L13	15.9/9.47	19/9	gi|126643019	TRB	161	4	42	-
443	50S ribosomal protein S8	14.2/10.05	15/10	gi|169632364	TRB	301	4	38	-
445	Host factor I for bacteriophage Q β-replication	16.8/6.9	<15/6	gi|169633286	NTM	98	2	12	-
450	50S ribosomal protein L23	11.5/9.49	<15/9	gi|126643093	TRB	139	2	35	-
451	50S ribosomal protein S10	11.7/9.3	<15/9	gi|126643096	TRB	474	5	44	-
452	50S ribosomal protein L25	11.5/9.62	<15/9	gi|126640884	TRB	305	2	36	-
453	50S ribosomal protein L24	11.2/9.89	<15/9	gi|193078472	TRB	521	10	55	-

All the proteins were identified from MASCOT files with NCBInr. All protein spots were identified as proteins from *Acinetobacter baumannii* ATCC 17978 proteome, except for spots 2, 42, 46, 47, 99, 149, 173, 182, 204, 206, 229, 314, 324, 362, 364, 404, 412, 432, 443, 445 which were identified with proteins from other *Acinetobacter* baumannii strains.

a) The protein class is abbreviated as follows: ATM, amino acid transport and metabolism; CDCP, cell division and chromosome partitioning; CTM, carbohydrate transport and metabolism; CEOM, cell envelope biogenesis and outer membrane; CoE, coenzyme metabolism; D, Defence; EPC, energy production and conversion; GF, general function predicted only; Mult., multifunctional; NTM, nucleotide transport and metabolism; PTC, postranslation modification, protein turnover, chaperones; RPS, RNA and protein synthesis; RR, proteins involved in redox reactions; S, signalling; TRB, translation, ribosomal structure and biogenesis; Trans., transcription.
b) Matched peptides (information obtained from MASCOT analyses).
c) % coverage (information obtained from MASCOT analyses).
d) Information concerning the pAs was obtained from Table 1, from Smith *et.al.* 2007 [9]. pAs general function: Drug resistance pAs: 17, 18, 23, 22; examples of pAs with other predicted function, such as metabolism pA 24, iron transport pA 21, amino acid metabolisms pA 11 and others. pAs with possible role in virulence: 1, 4, 11, 17,18, 20, 21, 23, 22.

* Protein with the same identification number referred in Kwon *et.al.* 2009 [15].
** Protein with the same identification number referred in Fernández-Reyes *et.al.* 2009 [47].
*** Protein with same identification number referred in both Kwon *et.al.* 2009 [15] and Fernández-Reyes *et.al.* 2009 [47].
