# Supplementary material for: 2-DE analysis indicates that Acinetobacter baumannii displays a robust and versatile metabolism
Source: Proteome Sci. 2009 Sep 28;7:37. doi: 10.1186/1477-5956-7-37 (PMC2761859; doi:10.1186/1477-5956-7-37)
Supplement: Additional file 2 — Table S2 - MALDI-TOF/TOF identification of Acinetobacter baumannii membrane protein spots. Identified proteins are listed with 2-DE spot numbers, protein description, theoretical Mr and pI, in gel Mr and pI, accession numbers, functional class, values resulting from Mascot data (score, number of matched peptides and percentage coverage) and information concerning the respective pAs. [file 1477-5956-7-37-S2.PDF]

Table S2. MALDI-TOF/TOF Identification of *Acinetobacter baumannii* membrane protein spots. The databases were queried with resulting Mascot data files.

Spot no.	Identified protein	Theoretical kDa/p*I*	In gel kDa/p*I*	Acession no.	Class [a)]	Score	Match pept. [b)]	(%) Cov. [c)]	pA no. [d)]
459	Putative Omp copper receptor (OprC)	67.7/6.08	64/6	gi|126640271*	T	69	1	3	-
460	Putative long-chain fatty acid transport protein	49.9/4.88	52/4	gi|126642803*	T	124	3	9	-
461	Putative signal peptide	52.8/5.05	55/3	gi|126641049	S	205	3	15	-
462	F0F1 ATP synthase α-subunit	55.5/5.29	56/4	gi|162286757*	EPC	223	6	14	-
463	Putative protein (DcaP-like)	44.8/5.79	53/6	gi|126642784*	T	190	5	18	-
464	Putative protein (DcaP-like)	44.8/5.79	53/6	gi|126642784*	T	741	9	34	-
465	Putative Omp (OprD)	46.4/5.22	45/4	gi|126640296*	T	280	5	20	-
466	Putative glucose-sensitive porin (OprB-like)	43.0/5.29	46/4	gi|126642873*	T	549	9	34	-
467	Omp38 precursor (OmpA)	37.3/5.13	39/4	gi|126642864*	T	690	12	30	-
468	Omp 33-36 kDa	32.1/4.77	32/3	gi|193078641	T	465	5	28	-
469	Omp CarO precursor	24.4/4.70	28/3	gi|126642573*	T	145	2	20	-
470	Putative Omp (Omp 25)	25.5/4.47	27/3	gi|72535027	T	479	8	52	-
471	Putative Omp	27.6/4.59	28/3	gi|126643324*	T	506	7	40	-
472	Omp CarO precursor	24.4/4.70	26/3	gi|126642573*	T	308	4	35	-
473	Omp W	20.0/5.09	22/4	gi|126640380*	T	435	7	38	-
474	Omp W	20.0/5.09	22/4	gi|126640380*	T	356	6	44	-
475	Putative peptidoglycan-binding LysM	16.9/5.02	22/4	gi|193076594	S	163	4	32	-
476	Putative peptidoglycan-binding LysM	11.9/4.73	21/4	gi|126640876	S	120	3	24	-
478	Glutathione peroxidase	20.2/4.88	26/4	gi|126640260	RR	98	2	14	-
480	Putative protease	19.1/5.95	23/6	gi|126642815	ATM	78	10	68	-
481	Bacterioferritin	18.1/5.02	18/4	gi|126640856*	T	150	4	26	5

Table S2. continued

Spot no.	Identified protein	Theoretical kDa/p*I*	In gel kDa/p*I*	Acession no.	Class [a)]	Score	Match pept. [b)]	(%) Cov. [c)]	pA no. [d)]
483	F0F1 ATP synthase β-subunit	50.3/5.03	55/3	gi|162286755**	EPC	75	14	44	-
484	F0F1 ATP synthase β-subunit	50.3/5.03	55/3	gi|162286755**	EPC	360	7	18	-
485	F0F1 ATP synthase α-subunit	55.5/5.29	55/4	gi|162286757*	EPC	522	8	24	-
487	Putative protein (DcaP-like)	44.8/5.79	53/6	gi|126642784*	T	109	2	6	-
488	Putative protein (DcaP-like)	44.8/5.79	53/7	gi|126642784*	T	308	4	15	-
489	Elongation factor Tu	41.5/4.99	53/4	gi|162286746	TRB	115	3	12	-
490	F0F1 ATP synthase γ-subunit	12.3/4.92	55/3	gi|126640257	EPC	61	1	11	-
491	Aspartate carbamoyltransferase catalytic subunit	36.8/6.15	39/7	gi|162286738	ATM	141	4	15	7
492	Elongation factor Ts	30.8/5.27	36/4	gi|126642362*	TRB	305	5	21	-
493	Two-component response regulator	28.8/5.91	32/7	gi|193078590	S	64	2	12	-
494	Hypothetical protein A1S_0015	53.3/6.08	29/8	gi|126640130	U	125	3	7	-
495	NADH dehydrogenase I chain B	25.8/6.17	28/8	gi|193076535	RR	100	3	9	-
496	Omp38 precursor (OmpA)	37.3/5.13	23/8	gi|126642864*	T	79	2	7	-
497	50S ribosomal protein L10	18.1/5.62	<15/6	gi|193076142	TRB	183	3	21	-
498	Universal stress protein	11.5/4.83	<15/6	gi|126642117	D	125	2	40	18
499	F0F1 ATP synthase γ-subunit	12.3/4.92	<15/4	gi|126640257*	EPC	61	1	11	-

All the proteins were identified from MASCOT files with NCBInr. All protein spots were identified as proteins from *Acinetobacter baumannii* ATCC 17978 proteome.

a) The protein class is abbreviated as follows: ATM, amino acid transport and metabolism; D, Defence; EPC, energy production and conversion; RR, proteins involved in redox reactions; S, signalling; T, transport.
b) Matched peptides (information obtained from MASCOT analyses).
c) % coverage (information obtained from MASCOT analyses).
d) Information concerning the pAs was obtained from Table 1, from Smith *et.al.* 2007 [9]. pAs general function: Drug resistance pAs: 18; pA with other predicted function, amino acid metabolism pA 5 and 7. pAs with possible role in virulence: 18.

* Protein with NCBI identification number referred in Kwon *et.al.* 2009 [15].

** Protein with NCBI identification number referred in Fernández-Reyes *et.al.* 2009 [47].
