# Supplementary material for: 2-DE analysis indicates that Acinetobacter baumannii displays a robust and versatile metabolism
Source: Proteome Sci. 2009 Sep 28;7:37. doi: 10.1186/1477-5956-7-37 (PMC2761859; doi:10.1186/1477-5956-7-37)
Supplement: Additional file 3 — Table S3 - MALDI-TOF/TOF identification of Acinetobacter baumannii proteins with the respective matched peptide sequences. Complementary results of the MALDI-TOF/TOF and MASCOT analyses. For each protein the number and the sequences of matched peptides and corresponding NCBI identifier are provided. [file 1477-5956-7-37-S3.PDF]

Table S3. MALDI-TOF/TOF identification of *Acinetobacter baumannii* proteins with the respective matched peptide sequences.

Spot no.	Identified protein	Accession no.	Matched peptides	Matched peptides (Ion score)
1	Chaperone Hsp 70	gi|126642981	8	R.RYEDQAVQK.D (52)<br>K.KTAEDYLGETVTEAVITVPAYFNDAQR.Q (17)<br>K.TAEDYLGETVTEAVITVPAYFNDAQR.Q (61)<br>R.AKLEGLVADLVAR.T (114)<br>K.VQEFFGREPR.K (54)<br>K.SQVFSTAADNQPAVDISVYQGER.K (94)<br>K.SQVFSTAADNQPAVDISVYQGERK.M (36)<br>K.LLGNFQLGDIPPAPR.G (103)
2	Heat shock protein 90	gi|169634585	3	R.RAGLDASEGVR.W (43)<br>R.FVQGVVDSADLPLNVSR.E (111)<br>R.LVDSPACLVTSEGELSPQLIR.M (65)
3	30S ribosomal protein S1	gi|126641617	3	K.GGFTVDIGPVR.A (34)<br>R.ANPWEEFAK.S (62)<br>K.QLNSDPFNDFLAANER.G (71)
5	Elongation factor	gi|126640918	7	R.LAFVNKMDR.T (60)<br>R.IVQMHANERQDIDEIR.A (54)<br>R.LAKEDPSFR.V (41)<br>R.EFGVEANIGKPMVAYR.E (94)<br>K.TVEQEGKFVR.Q (83)<br>K.EFFGAVDKGIQER.M (124)<br>K.MAGSYAFR.D (38)
6	Polynucleotide phosphorylase/ polyadenylase	gi|126640439	4	R.ASEAETLISR.L (64)<br>R.DNILSGKPR.I (75)<br>R.ALDVQVGVLER.A (71)<br>R.QITEETKAAIDIEDNGTVR.V (50)
8	Glutamine synthetase	gi|126642389	3	K.DYESGNSGHRPR.V (32)<br>R.VKGGYFPVPPVDSAQDMR.A (37)<br>K.GGYFPVPPVDSAQDMR.A (63)
9	Phosphoenolpyruvate carboxykinase	gi|126642702	3	K.YIVHYPETR.E (55)<br>R.DAEGNFVWPGFGQNMR.V (89)<br>R.VLEWIIDRCEGR.A (29)
21	Malic enzyme	gi|126642378	5	K.IACVHAIAR.M (52)<br>R.IAYAEGEDERVLR.A (27)<br>R.AVQIAVDEDLAKPILVGR.T (81)<br>R.KGITVEYAQR.E (70)<br>K.GITVEYAQR.E (44)
33	Trigger factor spetum formation molecular chaperone	gi|126640548	4	K.QVEKPKLPEIDAEFLK.I (37)<br>K.SMLPDELFKEQAER.S (87)<br>R.AQIEAVVLEDQVVDHILASAK.V (157)<br>K.AVSYEDLLKEQQAR.R (32)
34	Chaperone Hsp60	gi|126642698	6	K.SVTAGMNPMDLKR.G (58)<br>K.VGKEGVITVEEGSGFEDALDVVEGMQFDR.G (47)<br>K.EGVITVEEGSGFEDALDVVEGMQFDR.G (40)<br>K.ITVSKENTVIVDGAGDAAAIAER.V (115)<br>R.AAVEEGVVAGGGVALVR.A (177)<br>R.AVNALEGLKGANEDQTAGINILR.R (74)
35	Chaperone Hsp60	gi|126642698	5	R.GIDIAVKTVVENIR.S (93)<br>R.GIDIAVKTVVENIR.S (43)<br>K.ITVSKENTVIVDGAGDAAAIAER.V (140)<br>R.AAVEEGVVAGGGVALVR.A (140)<br>R.AVNALEGLKGANEDQTAGINILR.R (132)
36	Chaperone Hsp60	gi|126642698	1	R.AAVEEGVVAGGGVALVR.A (135)

Table S3. Continued.

Spot No.	Identified protein	Accession no.	Matched peptides	Matched peptides (Ion score)
38	Phosphoglycerate mutase III cofactor independent	gi|126640323	8	R.VLYQDFTR.I (75)<br>R.TGAFFEHEVLVDAVEK.A (83)<br>R.YFAMDRDNR.W (25)<br>R.WDRVEQAYR.L (19)<br>R.LLTEGEAVR.T (58)<br>R.TANTAVEGLELAYAANENDEFVKATR.I (34)<br>K.VQDGDSVVFMNFR.A (87)<br>R.AFVEKDFAGFER.K (102)
40	Phosphoglyceromutase	gi|126640323	2	R.VLYQDFTR.I (31)<br>R.AFVEKDFAGFER.K (58)
42	NAD-linked malate dehydrogenase, Rossman fold	gi|169797658	2	K.GSAFTQEER.S (39)<br>K.VIVITDGER.I (67)
46	Urocanate hydratase	gi|169634859	5	R.YVDEQATDLDDALAR.I (41)<br>K.MGVPTFDYGNNIR.Q (31)<br>R.SGELSAPIVIGR.D (44)<br>R.DHLDSGSVASPNR.E (21)<br>R.VLTNDPATGVMR.H (33)
47	Urocanate hydratase	gi|169634859	8	K.SWLTEAPLR.M (39)<br>K.ELVVYGGIGR.A (90)<br>R.YVDEQATDLDDALAR.I (103)<br>K.MGVPTFDYGNNIR.Q (84)<br>R.ISFQGLPAR.I (50)<br>K.LGLAFNEMVR.S (63)<br>R.SGELSAPIVIGR.D (83)<br>R.VLTNDPATGVMR.H (53)
48	Hypothetical protein A1S_2187	gi|126642232	2	R.VGKAEGEADLFPR.T (93)<br>R.TFNTQLNKAQDLR.Y (74)
54	F0F1 ATP synthase β-subunit	gi|162286755	4	K.IYDALQVDGTETTLEVQQQLGDGVVR.T (45)<br>R.DVLLFVDNIYR.Y (63)<br>R.DIASSGIYPAIDPLDSTSR.Q (69)<br>R.QLDPLVVGQEHYEIAR.A (36)
58	F0F1 ATP synthase α-subunit	gi|162286757	5	R.VLEVPVGPELLGR.V (88)<br>K.SVDTMIPVGR.G (65)<br>K.QSTIANVVR.K (69)<br>R.DRGEDALIIYDDLSK.Q (43)<br>R.EAYPGDVFYLHSR.L (94)
59	F0F1 ATP synthase α-subunit	gi|162286757	10	R.VLEVPVGPELLGR.V (62)<br>R.VVDALGNPIDGKGPIDAK.L (30)<br>K.LTDAVEKVAPGVIWR.Q (42)<br>R.QSVDQPVQTGYK.S (35)<br>K.SVDTMIPVGR.G (50)<br>R.EAYPGDVFYLHSR.L (45)<br>R.VSAEYVEKFTNGAVTGK.T (49)<br>R.ELAAFAQFASDLDEATR.K (53)<br>R.ELAAFAQFASDLDEATRK.Q (57)<br>K.AGIESFKATQTY.- (38)
64	Serine hydroymethyltranferase	gi|126642348	3	K.TYNAVQYGLNAETGEIDYEEVER.L (36)<br>K.NAQAMAEVFIAR.G (40)<br>R.SPFVTSGIR.I (34)
66	Putative protein (DcaP-like)	gi|126642784	2	K.AGADVNLYGFVR.G (31)<br>R.STLAYGAQFSDDGTDYAR.L (49)

Table S3. Continued.

Spot No.	Identified protein	Accession no.	Matched peptides	Matched peptides (Ion score)
67	Serine hydroymethyltranferase	gi|126642348	3	R.MIVAGFSAYSR.V (31)<br>K.LQSAVFPGNQGGPLMHAIAAK.A (83)<br>K.NAQAMAEVFIAR.G (53)
75	Gamma-glutamyl phosphate reductase	gi|126640560	3	R.SNQLDSALLDRLELTPAR.F (22)<br>K.VAGLPEHSVQVIETSDR.G (62)<br>R.GPVGLEGLTSQK.W (48)
80	Dihydrolipoamide dehydrogenase	gi|126642748	4	R.QLDPLVVGQEHYEIAR.A (86)<br>K.AYAEGLLAEDSGIKLTER.G (36)<br>R.GLVEVNDHCATSVEGVYAIGDLVR.G (66)<br>K.TGQFGFAVNGR.A (41)
85	Diaminobutyrate-2-oxoglutarate aminotransferase	gi|126642490	2	K.LATEFPCIGNVR.G (68)<br>R.GLMIGVEIVDER.K (42)
88	Cell division protein FtsZ	gi|126643338	2	R.GLGAGANPEVGQVAAEESR.E (56)<br>R.NTVSHTSTQSAQSVDEDDVPAINKR.Q (53)
92	Enolase	gi|126641943	3	R.ACAPSGASTGSR.E (25)<br>R.AGAEVFHSLK.S (80)<br>K.QGLNTAVGDEGGFAPNLR.S (90)
94	Dihdrolipoamide succinyltranferase component of 2-oxoglutarate dehydrogenase complex (E2)	gi|126642747	4	R.NETVSDQAPAVR.K (45)<br>R.KALTESGIAASDVQGTGR.G (81)<br>K.ALTESGIAASDVQGTGR.G (66)<br>R.ITKEDVANHQAKPAANVTPLSVAVGER.I (18)
95	Dihdrolipoamide succinyltranferase component of 2-oxoglutarate dehydrogenase complex (E2)	gi|126642747	5	R.KALTESGIAASDVQGTGR.G (110)<br>K.ALTESGIAASDVQGTGR.G (66)<br>R.ITKEDVANHQAKPAANVTPLSVAVGER.I (12)<br>K.EDVANHQAKPAANVTPLSVAVGER.I (17)<br>R.DTDRMSYAEVEAGIAAYAAK.A (40)
96	Histidinol dehydrogenase	gi|126642747	3	K.AAFDGLNQEIR.E (50)<br>R.VDKITGPGNR.F (36)<br>K.TADVLAVQENLDAHAR.S (43)
99	Beta-ketoacyl-ACP synthase I (3-oxoacyl-[acyl-carrier-protein] synthase I)	gi|169634061	6	R.FNPTYAELNFK.S (86)<br>K.SHVSAAAEQDFDNIDR.K (101)<br>K.SHVSAAAEQDFDNIDRK.L (67)<br>R.KVGPFFVPR.N (68)<br>K.VGPFFVPR.N (40)<br>K.YNDTPETASRPYSK.D (64)
100	Protein chain elongation factor EF-Tu	gi|162286753	3	R.GITINTSHVEYDSPTR.H (58)<br>R.ELLSTYDFPGDDTPVIR.G (69)<br>R.AGENCGILLR.G (44)
103	Aspartate aminotransferase A	gi|126642544	2	K.ITPEQLEAAITPNTR.L (101)<br>K.AYAMTGWR.I (57)<br>R.RHDLVVNGLNDIK.G (22)<br>R.ISYATADEVLVDALAR.I (171)
104	Methionine adenosyltranferase	gi|126641564	4	R.ETDVLMPAPISYAHR.L (40)<br>R.SGALPWLRPDAK.S (53)<br>R.KIIVDTYGGMAR.H (41)<br>K.QTAAYGHFGR.E (49)
105	Hypothetical protein A1S_2277	gi|126642318	4	K.TSNLNVIGEVELR.I (70)<br>R.IHHQFLVSENTR.K (96)<br>K.QIYAHQQTLAQCR.Q (72)<br>R.QWLDAHYPGVER.V (65)

Table S3. Continued.

Spot No.	Identified protein	Accession no.	Matched peptides	Matched peptides (Ion score)
108	Threonine synthase pyridoxal-5´-phosphate-dependent enzyme	gi|126640331	7	K.AIICASTGNTSAAAAAYAAR.A (62)<br>R.GNFDDGMR.L (44)<br>K.EVADQAPVTIVNSINPYR.L (51)<br>K.TIAYEIVEALGR.A (111)<br>R.GAPVEKPETVATAIR.I (84)<br>R.IGNPQSWNHAK.A (49)<br>K.GWFDELQDSEILEAQR.L (77)
110	Succinylornithine transaminase	gi|126643147	1	R.FTPSLIIPEQDIDEGIAR.L (61)
114	Hypothetical protein A1S_3388	gi|126643395	2	R.TIQTEYGSFELYR.Y (50)<br>K.LNKADGEPAWNLDR.A (26)
115	WecE protein	gi|126640168	2	K.TYNLDSEKLEAAITPR.T (41)<br>K.LEILDDEMQAR.Q (64)
120	Imidazolonepropionase	gi|126643410	3	R.EASEEQLLNSALKR.I (95)<br>R.ETQYPPIESLIK.H (34)<br>R.VVQHGQEVIF.- (65)
123	Phospho-2-dehydro-3-heoxyheptonate aldolase	gi|126641709	2	K.GLINDPDMNDSFNIEKGLR.I (18)<br>K.GNPYAHVVLR.G (59)
124	D-3-phophoglycerate dehydrogenase	gi|126643167	3	R.GIPVFNAPYSNTR.S (37)<br>K.EGAIFLNAAR.G (47)<br>K.ANGEEFQSPLR.G (52)
132	Branched-chain amino acid transferase	gi|126642928	2	K.VPYDQAALEQAQIDVVR.E (36)<br>K.DLGYEVVER.R (66)
134	Succinyl-CoA synthetase β-chain	gi|126642749	1	K.SKEDVIEFANNIIGTR.L (95)
135	Phosphoglycerate kinase	gi|126641588	2	K.LFTDYLDGVEVEAGQVVLLENVR.F (42)<br>K.SLYEADLVETAK.Q (39)
137	Succinyl-CoA synthetase α-chain	gi|126642750	2	R.LVGPNCPGVITPGECK.I (47)<br>K.SNVTKPVVGYIAGVTAPK.G (17)
138	Fructose-1,6-bisphosphatase	gi|126642631	2	R.GGIFLYPYDLK.D (21)<br>R.ILEIEPTELHQR.V (40)
140	Outer membrane protein omp38 precursor	gi|126642864	6	K.YDFDGVNR.G (42)<br>R.GTSEEGTLGNAGVGAFWR.L (107)<br>K.VAEKLSEYPNATAR.I (49)<br>K.LSEYPNATAR.I (40)<br>R.IEGHTDNTGPR.K (59)<br>K.SALVNEYNVDASR.L (67)
145	DNA-directed RNA polymerase subunit alpha	gi|158513671	8	K.VILEPLER.G (54)<br>K.QGPGDITAADLR.L (34)<br>R.LQLDASYSPIKR.V (79)<br>R.VSYTVENAR.V (30)<br>R.EEVDPILLRPVDDLELTVR.S (23)<br>K.AENIYYIGDLVQR.T (70)<br>R.LENWPPASLR.M (64)<br>R.MDDRFAYR.S (24)
149	Outer membrane protein omp38 precursor	gi|126642864	4	K.YDFDGVNR.G (40)<br>K.LSEYPNATAR.I (36)<br>R.IEGHTDNTGPR.K (52)<br>K.SALVNEYNVDASR.L (127)

Table S3. Continued.

Spot No.	Identified protein	Accession no.	Matched peptides	Matched peptides (Ion score)
154	Succinyl-CoA synthetase alpha chain	gi|126642750	9	R.YLETNGNGTR.L<br>R.LVGPNCPGVITPGECK.I<br>K.IGIMPGHIHQPGR.I<br>R.SGTLTYEAVAQTTK.L<br>K.SNVTKPVVGYIAGVTAPK.G<br>K.RMGHAGAIISGGQGTAEEK.F<br>R.MGHAGAIISGGQGTAEEK.F<br>R.SPAELGSTMLQVLK.E<br>R.SPAELGSTMLQVLKEK.G
156	Fructose-1,6-bisphosphate aldolase, class II	gi| 126641589	6	R.AIMLAADATNSPVIVQASAGAR.K (54)<br>R.KYAGAPFLR.H (41)<br>R.SIQLGFSSVMMDGSLGADGKTPTTYDYNVDVTR.Q (19)<br>K.VINEFGGNIGETYGVPVEQLVEAIKHGVR.K (38)<br>R.RFMAENPAEFDPR.K (32)<br>R.FMAENPAEFDPR.K (28)
158	Succinyl-CoA synthetase alpha chain	gi| 126642750	2	R.LVGPNCPGVITPGECK.I (65)<br>K.SNVTKPVVGYIAGVTAPK.G (63)
171	Thioredoxin reductase	gi| 126640925	1	K.FMGQGVSACATCDGFFYK.N (99)
172	Putative flavohemoprotein	gi| 126643100	1	R.SFEITQIDPLESGKR.F (56)
174	Putative intercellular/amidase	gi| 193078218	4	K.TDYDGTTYPTPYAGNKK.V (41)<br>K.QEQVVLDTFQK.Y (54)<br>K.DYQICVFPDSLDK.G (42)<br>K.ILNTGITGQCHR.D (56)
176	Dihydrodipicolinate synthase	gi| 126643430	6	R.IPIIAGTGANSTR.E (18)<br>R.LAEIPNIVGIKDATGDVPR.G (57)<br>K.AMSEVCAVAIAKDEQQAK.T (19)<br>K.WALHEMGLIDTGIR.L (319<br>R.LPLTPLAEQYR.E (37)<br>R.LPLTPLAEQYR.E (29)
178	Elongation factor Ts	gi| 126642362	10	K.KALTEANGDIELAIDNLR.K (71)<br>K.ALTEANGDIELAIDNLR.K (109)<br>K.LADGQSVEEAR.I (75)<br>K.IGENIQVR.R (63)<br>K.IVEGEQLAIYKHGLK.I (72)<br>K.MVTGSVEKYLNEVALDR.Q (94)<br>K.YLNEVALDR.Q (79)<br>K.ATGTNVANFVR.F (73)<br>K.KAELSFAEEVAAAQAAAK.- (72)<br>K.AELSFAEEVAAAQAAAK.- (90)
180	Malate dehydrogenase	gi| 152032571	10	R.VAVTGAAGQIGYSLLFR.I (64)<br>K.VAFKDADYALLVGSRPR.G (24)<br>K.DADYALLVGSRPR.G (26)<br>R.ADLLKVNGEIFIGQGQALNEVASR.D (50)<br>K.VNGEIFIGQGQALNEVASR.D (98)<br>K.SAPDLPAKNFTAMLR.L (33)<br>K.AGVAVADIEKLTVWGNHSPTMYADYR.F (13)<br>K.IVQGLEIDEFSR.E (79)<br>K.IVQGLEIDEFSRER.I (34)<br>R.INFTLNELEEERAAIADMVK.- (21)
182	Subunit of cysteine synthase A and O-acetylserine sulfhydrolase A, PLP-dependent enzyme	gi| 169632185	2	R.IGAALIADAEKR.G (50)<br>K.TIVVILPDSGER.Y (22)

Table S3. Continued.

Spot No.	Identified protein	Accession no.	Matched peptides	Matched peptides (Ion score)
186	Cysteine synthase B	gi|126640646	2	K.LIMPGNSSQER.K (28)<br>R.AYGAELIEAPNMEAAR.D (38)<br>K.EQNPDIQIIGLQPSEGSNIAGIR.R (31)
193	NADH-dependent enoyl-ACP reductase	gi|126640605	3	K.LSIAYGIAQALHR.E (44)<br>R.QGCLLTLTYQGSER.V (21)<br>R.YLASSLGVDGIRVNAISAGPIR.T (12)
192	Hypothetical protein A1S_1833	gi|193077447	3	K.DGVDIFYKDWGPR.D (69)<br>K.NAIHIGHSTGGGEVVR.Y(13)<br>K.EVFDDLQNQVLTNR.A(119)
194	Putative acetyl-CoA carboxylase, beta subunit	gi|126642893	2	K.FVDSKPYPDR.M (40)<br>R.ETLEEPFQR.A (60)
195	NADH_dependent enoyl-ACP reductase	gi|126640605	9	M.TQGLLAGKR.F (44)<br>R.EGAELAFTYPNEK.L (70)<br>R.EGAELAFTYPNEKLK.K (47)<br>K.RVDEFAEQFGSK.L (49)<br>R.VDEFAEQFGSK.L (31)<br>R.AAKPLLQAR.Q (39)<br>R.QGCLLTLTYQGSER.V (78)<br>R.YLASSLGVDGIR.V (68)<br>R.VNAISAGPIR.T (48)
200	50S ribosomal protein L1	gi|126640372	4	K.FKESLDISVNLGVDPR.K (29)<br>R.VAVFAQGAQAEAAK.E (93)<br>K.VGTVTPDVAGAVK.N (43)<br>K.AGIIHAAIGQVGFDAAAIR.Q (162)
202	Putative outer membrane protein	gi|126643304	3	K.GEAYVPTPYLPVYASATYNHTDVDGK.N (16)<br>K.LSVGATFVGNDGEADIK.D (42)<br>K.SSYDTQTIGLNAK.F (33)
204	Conserved hypothetical protein	gi|169797441	2	R.TYEEMEEMER.N (33)<br>K.EAFFHAYMTEGLAIGER.E (31)
206	Electron transfer flavoprotein alpha-subunit	gi|169632682	3	M.SILVIADHNNQTLNGATLNVVAAAQK.I (55)<br>K.IGGDITVLVAGSGAQAVADAAAK.V (28)<br>K.VLDPLADKLGAAQGASR.A (59)
207	Electron transfer flavoprotein α-subunit	gi|126642662	6	K.RPIYAGNAIATVQSDEAIIVGTVR.G (7)<br>R.GTAFDPVAAEGGSAAVETVGEVKDAGVSK.F (49)<br>K.FVSEEIVKLDRPELTAAR.I (49)<br>R.IVVSGGRGVGSGENYHK.V (35)<br>K.VLDPLADKLGAAQGASR.A (137)<br>R.AAVDAGFVPNDFQVGQTGK.I (62)
208	Response regulator (activator) in two-component regulatory (OmpR family)	gi|126640806	3	R.TEDMDQVLGLEMGADDYVAKPVQPR.V (73)<br>R.IEFDDLVIDNGGR.S (45)<br>R.GIEYDGQDR.S (28)
211	2,3,4,5-tetrahydropyridine-2-carboxylate N-succinyltransferase	gi|126642574	2	R.QAVEEAIAGLDNGTLR.V (63)<br>K.LNDNKPIESCDLR.F (26)
212	Adenylate Kinase	gi|126641073	5	R.IAQPDCVNGCIFDGFPR.T (56)<br>K.EGISIDHVIEIDVPDEEIVKR.L (46)<br>R.LASYHTETEQLVGFYQGR.A (73)<br>R.AASGENAPTYDKLDGLR.T (59)<br>R.TIEDVQKDLFNILDK.- (59)
214	2,3,4,5-tetrahydropyridine-2-carboxylate N-succinyltransferase	gi|126642443	4	R.ANFTAADCPSEIR.Q (33)<br>R.QAVEEAIAGLDNGTLR.V (59)<br>K.LNDNKPIESCDLR.F (53)<br>K.FSGWTEEQFK.A (56)

Table S3. Continued.

Spot No.	Identified protein	Accession no.	Matched peptides	Matched peptides (Ion score)
222	Acetyl-coenzyme A carboxylase carboxyl transferase	gi|126640675	2	R.FDGQPVMVIGQHR.G (104)<br>K.QALDELLPMDANER.C (83)
229	30S ribosomal protein S2	gi|169632956	2	R.DLLQAGAHFGHQTR.F (97)<br>R.AGQPYVDHR.W (31)
233	Tryptophan synthase alpha chain	gi|126388778	3	K.QHDMDQIFLLAPTSTDQR.I (75)<br>R.IQHVANQASGFIYYVSLK.G (17)<br>K.SFATLAADEAVEQTVNKVK.E (40)
234	1-(5-phophoribosyl)-5-[(5-phosphoribosylamino)methylideamino] imidazole-4-carboxamide isomerase	gi|126643246	4	K.AQPELPIQIGGGIR.S (78)<br>K.AVQEPEFVEEACKR.F (69)<br>K.GQPGILGAITGR.A (61)<br>R.EAQLLLDENRL.- (40)
235	Elongation factor P	gi|126642455	4	R.GDTSGGGGKPAKLETGAVVR.V (73)<br>R.VPLFVQQEESVR.V (122)<br>R.VPLFVQQEESVRVDTR.T (55)<br>R.TGEYLERA.- (47)
236	Acetoacetyl-CoA transferase alpha subunit	gi|126641777	7	-.MISSYVGENKEFER.Q (37)<br>R.QYLNGELEVELTPQGTLAEK.L (67)<br>R.QYLNGELEVELTPQGTLAEKLR.A (17)<br>K.NYILEESLTADVALVK.A (50)<br>R.KTAQNFNPECAMAGK.I (25)<br>K.TAQNFNPECAMAGK.I (30)<br>R.IVLNASPEKR.I (18)
242	Acetyl-coenzyme A carboxylase carboxyl transferase	gi|126640675	1	R.FDGQPVMVIGQHR.G (120)
244	Cytidylate kinase	gi|126641616	2	R.AFAQTPGLVADGR.D (35)<br>K.INDILANIQAR.D (58)
247	Superoxide dismutase	gi|126642383	2	K.IDEAFGSYEK.F (92)<br>K.LAGQPAGVEK.- (45)
248	Ribosome releasing factor	gi|126642019	1	R.TLIVQPFER.T (62)
251	Putative protease	gi|193078237	3	K.NGEHIATAIHDFEGEQTYSEK.R (49)<br>K.NGEHIATAIHDFEGEQTYSEKR.G (122)<br>R.VVEIVREFDR.V (49)
254	Putative oxidoreductase	gi|126641974	3	R.EAVKHSPSAFNSQSSR.V (98)<br>R.AQLVFGSVEAPAGEK.A (115)<br>K.AFISDEDRFK.T (32)
257	Hypothetical protein A1S_0484	gi|126640557	3	R.TAAGLAAIER.A (69)<br>R.ASQLADDIVVGVGTITKPEHCAQAK.K (101)<br>K.AFYGPFPNLR.F (77)
259	Xanthine phosphoribosyltransferase	gi|126643050	3	K.SFQPGRDLLLEK.G (31)<br>K.GYRVESLAR.V (30)<br>R.VQSLADGTVTFVKE.- (28)
265	Outer membrane protein CarO precursor	gi|126642573	2	K.NDIAPYLGFGFAPK.I (34)<br>K.QGTFVNAAGGNADADLR.A (45)
267	Hps 24 nucleotide exchange factor	gi|126642980	3	R.TANAVYEAQKSVER.I (38)<br>K.ELLDSVDNLER.A (37)<br>K.ANEIGTVLQK.G (43)
268	Inorganic pyrophosphatase	gi|126640295	3	K.LIAVPHEKLSPLYK.D (53)<br>K.ISGWEGADVAK.A (51)<br>K.ISGWEGADVAKAEVLK.A (77)
269	50S ribosomal protein L4	gi|126643094	2	R.LVLVEEFAVAAPK.T (35)<br>R.ALIVTDAVDENLYLAAR.N (37)

Table S3. Continued.

Spot No.	Identified protein	Accession no.	Matched peptides	Matched peptides (Ion score)
270	50S ribosomal protein L4	gi|126643094	4	R.EFNEALVHQVVTAYLAGGR.Q (22)<br>K.TFAARPQDWSQK.V (26)<br>R.LVLVEEFAVAAPK.T (43)<br>R.ALIVTDAVDENLYLAAR.N (49)
271	Alkyl hydroperoxide reductase C22 subunit	gi|126641253	2	K.NFDVLIESEGLADR.G (45)<br>K.IQIVEINAGGIGR.D (82)
273	Alkyl hydroperoxide reductase C22 subunit	gi|126641253	3	K.NFDVLIESEGLADR.G (95)<br>K.IQIVEINAGGIGR.D (43)<br>K.EGEATLAPSIDLVGKI.- (59)
283	ATP-dependent Clp protease proteolytic subunit	gi|126640549	5	R.YCLENAR.V (41)<br>R.VMIHQPLGGFR.G (22)<br>R.GQASDIEIHAR.E (28)<br>R.LMAEHSGQDYDTIAR.D (16)<br>K.EYGLVDQVLSKRP.- (14)
285	Peptidyl-prolyl cis-trans isomerase precursor	gi|126642154	2	R.DAIENEADNGLSNDVGTIAMAR.T (57)<br>R.GYHADVPLENVVIESAK.I (46)
296	Transcription elongation factor	gi|126642719	5	-.MQRYPMTPEGK.I (23)<br>R.ITQAIAEAR.E (37)<br>R.EHGDLKENAEYHAAR.E (21)<br>R.EQQGFCEGR.I (42)<br>K.LGAAQVIDVKDLEQNGR.V (31)
297	Omp W	gi|126640380	6	K.FGGSVIAPSEDTTTALGVVK.A (118)<br>R.IKQLPPTITAK.Y (59)<br>K.YHFKNSTR.F (18)<br>K.EDFGVAGQIGFNFQPADAK.N (52)<br>K.NWGVFVDVR.Y (51)<br>R.YADISPEVTLTNGAK.F (51)
298	Putative peptidoglycan-binding LysM	gi|126640876	5	K.IILIVGNVDHVAQVDDQMTVATPEPESK.F (27)<br>K.IAKEFYGDANQYQK.I (51)<br>K.EFYGDANQYQK.I (48)<br>K.IFEANKPMLKDPDEIFPGQVLR.I (30)<br>K.DPDEIFPGQVLR.I (33)
299	Putative outer membrane protein W	gi|126640380	2	K.EDFGVAGQIGFNFQPADAK.N (25)<br>K.NWGVFVDVR.Y (72)
300	2,3,4,5-tetrahydropyridine-2-carboxylate N-succinyltransferase	gi|126642574	4	M.SQLSTIIEQAFEDR.A (37)<br>R.ANFTAADCPSEIR.Q (47)<br>R.QAVEEAIAGLDNGTLR.V (67)<br>K.LNDNKPIESCDLR.F (47)
305	Hypothetical protein A1S_0606	gi|126640673	1	K.NGEVVAQQIGAVPR.S (82)
307	Hypothetical protein A1S_2863	gi|193078285	5	K.SVFGGEFAMLSR.Y (38)<br>K.SVFGGEFAMLSR.Y (31)<br>R.YSDIPPNDGVTLSEAQK.N (82)<br>K.QLFDALSVNGK.I (60)<br>K.WMVNCQLDT.- (67)
310	Biotin carboxyl carrier protein of acetyl-CoA carboxylase (BCCP)	gi|126642055	2	R.RNPVVAAAGVALPAAPVAEAPVAK.T (27)<br>K.NGEVIQFGQPLFR.Y (81)

Table S3. Continued.

Spot No.	Identified protein	Accession no.	Matched peptides	Matched peptides (Ion score)
314	Nucleoside diphosphate kinase	gi|169634408	7	K.NHIGEIFAR.F (60)<br>K.HLSQADAEGFYAEHK.E (95)<br>K.HLSQADAEGFYAEHKER.G (139)<br>R.EILGATNPK.E (63)<br>K.EAAPGTIRADFAVSIDENAAHGSDSVASAER.E (33)<br>R.ADFAVSIDENAAHGSDSVASAER.E (158)<br>R.ADFAVSIDENAAHGSDSVASAER.E (70)
316*	50S ribosomal protein	gi|126640373	5	R.AFQGTQFDILNDNLVGPTILGFSTSEDDMGAAAR.L<br>R.LFEEFAK.T<br>R.LFEEFAKTNK.A<br>K.VYQGADVSVIANLPNQEK.A<br>K.ALTMLASVLQAPISK.L
317	Nucleoside diphosphate kinase	gi|126640569	2	R.ADFAVSIDENAAHGSDSVASAER.E (16)<br>R.EIAYFFADNEICPR.T (81)
318	50S ribosomal protein L9	gi|126642218	5	-.MDVILLQR.I (53)<br>K.AVAATEANTAAFEAR.R (142)<br>K.AVAATEANTAAFEARR.A (19)<br>K.AGDEGKLFGSIGTR.D (68)<br>R.DIADALTNAGLTVDR.A (67)
324				
328	Hypothetical protein A1S_0323	gi|126640405	3	R.MEIISGECR.V (19)<br>R.VKIADSNESELFR.A (56)<br>K.IADSNESELFR.A (48)
331	Putative DNA binding protein	gi|126640361	2	R.NKNNAEETWTGR.G (75)<br>K.NNAEETWTGR.G (57)
332	Putative stress protein (Usp)	gi|193077665	5	R.HIIVPVDGSEISLAAAR.Q (36)<br>K.ALCAEEGVDIETQIIQGEISSDGILK.A (147)<br>K.AVEELGSDLIVIGSHGRK.G (51)<br>K.LILGSFAQDVLNSTK.I (127)<br>K.IQVLVVKE.- (49)
333	Putative stress protein (Usp)	gi|193077665	6	R.HIIVPVDGSEISLAAAR.Q (30)<br>K.VKALCAEEGVDIETQIIQGEISSDGILK.A (24)<br>K.ALCAEEGVDIETQIIQGEISSDGILK.A (174)<br>K.AVEELGSDLIVIGSHGRK.G (51)<br>K.LILGSFAQDVLNSTK.I (106)<br>K.IQVLVVKE.- (58)
335	30S ribosomal protein S6	gi|126642216	2	R.YISQIKEADGQIHR.L (27)<br>R.EHAITEESLLAK.S (78)
342	50S ribosomal protein	gi|126640374	3	R.EATGLGLKEAK.D (29)<br>K.DLVEGAPQVLK.E (25)<br>K.KLEEAGATVTLK.- (86)
347	Co-chaperonin GroES	gi|126642699	2	M.SNIRPLHDR.V (35)<br>K.TAGGILLPGSAAEKPSQGEVIAVGNGQITDNGVR.A (20)
352	Nitrogen assimilation regulatory protein P-II 2	gi|126640311	2	R.QKGHTELYR.G (78)<br>R.IRTGETGPDAV.- (56)
353	Putative type III effector	gi|126640467	3	M.AQELLAQLQAGTAK.F (93)<br>K.FSDVLAYIEAR.Y (110)<br>R.YQHTPTAFQNGAQHNAATENQGSAK.V (9)
360	Putative toluene tolerance protein Ttg2F	gi|126640746	1	R.IVDDQFEGKR.T (61)
361	Chaperonin termination factor	gi|126640746	1	R.AAVEEGVVAGGGVALVR.A (79)<br>R.AVNALEGLKGANEDQTAGINILR.R (54)

Table S3. Continued.

Spot No.	Identified protein	Accession no.	Matched peptides	Matched peptides (Ion score)
362	Dihydrolipoamide dehydrogenase (Glycine oxidation Lfactor)	gi|169632626	5	M.SQQFDLVVIGGGPGGYEAAIR.A (179)<br>K.KVEFVPHEGETQILEPK.Y (144)<br>K.AYAEGLLAEDSGIKLTER.G (75)<br>R.GLVEVNDHCATSVEGVYAIGDLVR.G (65)<br>K.TGQFGFAVNGR.A (84)
364	Transcription termination factor Rho	gi|169634328	10	K.VNQINYDTPENSR.N (36)<br>R.VVDLVAPIGKGQR.S (25)<br>R.HVQVAEMVIEK.A (80)<br>K.DVVILLDSITR.L (69)<br>R.LMDEDKLR.K (53)<br>R.LMDEDKLR.K (46)<br>K.TNDDFFDQMK.R (40)<br>K.TNDDFFDQMK.R (29)<br>K.TNDDFFDQMKR.K (44)<br>K.TNDDFFDQMKR.K (26)
370	Hypothetical protein A1S_2371	gi|126642408	3	K.QLYLFNSR.N (39)<br>K.TASHGCIR.L (20)<br>R.LTNWDANDLGNK.V (34)
373	Putative short-chain dehydrogenase	gi|193077656	5	M.TDQYQAFTQSPIGK.F (42)<br>K.GITAQVVYVDEGAAANLESTLR.F (23)<br>R.DGAHVICLDVPQQQADLDR.V (13)<br>R.VNDYLLENDGLNANGR.I (34)<br>R.IVCVSSISGIAGNLGQTNYAASK.A (29)
379	Transcription termination factor	gi|169634328	3	R.SAAGSYLAGPDDIYVSPSQIR.R (21)<br>R.VVDLVAPIGKGQR.S (75)<br>K.TNDDFFDQMKR.K (44)
385	Mutarotase precursor	gi|126641015	2	K.NNGPNSLHSGNPGFDKR.V (53)<br>R.QADALALETQHFPDSPNQPAFPSTR.L (43)
389	Hypothetical protein A1S_3473	gi|126640111	3	R.ATHHLQPYK.F (75)<br>K.VAADGQKDAPEFNLR.G (47)<br>K.DAPEFNLR.G (40)
393	Heat shock protein Hsp40	gi|126643450	6	K.EASEAYEILSDSEKR.S (46)<br>K.TITFTAPAPCDVCDGK.G (33)<br>R.QQTLEVTIPAGVDNGDR.V (34)<br>R.DGQAGDLYVEVVVR.E (31)<br>R.EHEIFQR.D (33)<br>K.EIEIPTLEGR.V (31)
394	Lipoyl synthase	gi|162286727	2	R.IMTECPPDVFNHNIETVPR.L (40)<br>R.QYYGEPVPEVR.R (29)
400	Hypothetical protein A1S_0015	gi|126640130	3	K.LGFEYNAR.L (29)<br>R.LLANDIQPQHR.E (59)<br>R.GEGVEQSFTPDVGIR.H (87)
404	Electron transfer flavoprotein β-subunit	gi|169632681	7	R.VKPDNSGVDLTNVK.M (35)<br>K.MSINPFCEIAVEEAVR.L (35)<br>K.GTVSEIVVVSIGPK.E (70)<br>R.GILVETTDEIGALEVAK.I (31)<br>K.GVVDAEKPELILLGK.Q (52)<br>R.EIDGGLQTVELALPAIITTDLR.L (44)<br>K.SPADYGVTAGTK.L (42)
405	Putative hydrolase	gi|126642696	3	K.TGVDFDVNSEQAINTR.L (132)
406	30S ribosomal protein S2	gi|126642363	1	K.NLGIPVIGIVDTNSNPDNVDYVIPGNDDAIR.A (60)

Table S3. Continued.

Spot No.	Identified protein	Accession no.	Matched peptides	Matched peptides (Ion score)
407	50S ribosomal protein L1	gi|169634597	6	K.AIAAAVEANKVYTLEEAVQVLNSLPAAK.F (28)<br>K.VYTLEEAVQVLNSLPAAK.F (112)<br>K.FKESLDISVNLGVDPR.K (76)<br>R.VAVFAQGAQAEAAK.E (129)<br>K.EAGADVVGFDDLAESIQGGNLDFDVVIAAPDAMR.V (114)<br>K.AGIIHAAIGQVGFDAAAIR.Q (44)
408	Electron transfer flavoprotein beta-subunit	gi|126642663	7	-.MSINPFCEIAVEEAVR.L (113)<br>K.GTVSEIVVVSIGPKEAQEQIR.S (73)<br>R.SAMALGADRGILVETTDEIGALEVAK.I (34)<br>R.EIDGGLQTVELALPAIITTDLR.L (104)<br>R.EIDGGLQTVELALPAIITTDLRLNEPR.Y (13)<br>K.SPADYGVTAGTK.L (106)<br>K.TIKVEAPAER.K (36)
410	FKBP-type peptidyl-prolyl cis-trans isomerase	gi|126640161	3	K.AFVQGIHDAR.N (31)<br>R.LINGQVFDSSYK. (24)<br>R.LINGQVFDSSYKR.G (23)
412	30S ribosomal protein S4	gi|169632374	8	K.SGVKPFDVK.T (31)<br>K.QSEYSLQLR.E (39)<br>R.QFSNYYK.E (41)<br>K.AGDVIAVHEGAK.Q (76)<br>K.AGDVIAVHEGAKQQLR.I (22)<br>R.GIPAWIEVDHSK.L (35)<br>R.SDLPAEINESLIVELYSK.- (35)
418	50S ribosomal protein L3	gi|126643095	2	R.TQDATHGNSVSHR.V (83)<br>R.VTVQGLEIVSVDTER.S (59)
428	50S ribosomal protein L3	gi|126643095	7	R.IFTDAGVSVPVTVIEVDPNR.I (56)<br>R.IFTDAGVSVPVTVIEVDPNRITQIK.T (28)<br>K.TLETDGYQAVQVTTGER.R (73)<br>K.TLETDGYQAVQVTTGERR.E (41)<br>K.EFRVTEAELEGR.E (27)<br>R.VTVQGLEIVSVDTER.S (101)<br>K.GAIPGATGGDVIVRPTIKA.- (67)
432	50S ribosomal protein L6	gi|126643084	5	K.APVTVPNGVTVTQNGR.Q (97)<br>K.DAWMQAGTAR.A (54)<br>K.SANKQLLGQVAAEIR.A (78)<br>K.QLLGQVAAEIR.A (52)<br>R.YSDEVILRK.E (27)
437	50S ribosomal protein L5	gi|126643084	3	K.LLDGAVADMQLIAGQKPVVTLAR.K (25)<br>K.EQIVFPEIDFDKIDR.I (98)<br>R.GMDITITTTAR.T (35)
439	50S ribosomal protein L13	gi|126643019	4	K.TLSAKPAEVQHDWFVVDATGK.T (31)<br>R.HTEFPGGLK.E (36)<br>K.LVAHKPEEIFER.A (48)<br>K.VYAGSEHPHAAQQPQVLDI.- (49)
443	50S ribosomal protein S8	gi|169632364	4	M.SMQDTVADMLTR.V (101)<br>M.SMQDTVADMLTRVR.N (38)<br>K.VAIANVLQQEGYISNVEVAQEETK.S (130)<br>K.YFEGKPVIEMVKR.V (32)
445	Host factor I for bacteriophage Q β-replication	gi|169633286	2	K.HAISTVVPAR.N (24)<br>K.FEDGQDDENNR.- (74)
450	50S ribosomal protein L23	gi|126643093	2	R.IYQVLKGPVFSEK.A (64)<br>K.AYVTLKAGQDVEMADLGDTAESAAE.- (75)

Table S3. Continued.

Spot No.	Identified protein	Accession no.	Matched peptides	Matched peptides (Ion score)
451	50S ribosomal protein S10	gi|126643096	4	R.LIDQSAQEIVETAK.R (87)<br>R.LIDQSAQEIVETAKR.T (146)<br>R.TGAQVCGPIPMPTR.I (62)<br>R.TGAQVCGPIPMPTRIER.F (64)<br>R.FNVLTSPHVNKDAR.D (116)
452	50S ribosomal protein L25	gi|126640884	2	R.ESLVPAIIYGGNAEPVAVTLELR.E (191)<br>K.ALESNAFFEEVVEIK.V (113)
427	Putative signal peptide	gi|193077163	3	K.EFQEEAWFNAAKYPNITFK.S (30)<br>K.GITKPVVLDAVLNK.Q (73)<br>K.VPAIGFNATTSFK.R (102)<br>R.SDFGLGNYVPNVGDK.I (33)
453	50S ribosomal protein L24	gi|193078472	10	K.KGDQVIVIAGK.E (79)<br>K.GDQVIVIAGKEK.G (69)<br>K.GKQGTVLSVSEDR.V (38)<br>K.QGTVLSVSEDR.V (45)<br>R.VKVEGLNLVK.K (53)<br>K.VEGLNLVKK.H (56)<br>K.KHQKPNR.V (31)<br>K.ADRVGYQVIDGVK.T (23)<br>R.VGYQVIDGVK.T (80)<br>R.VGYQVIDGVKTR.V (47)
459	Putative Omp [e)] copper receptor (OprC)	gi|126640271	1	K.AGSAGFGFASEEQFNNIGR.N (69)
460	Putative long-chain fatty acid transport protein	gi|126642803	3	K.NFNFYAGPVLQTVK.G(58)<br>K.ETTGAGWLAGAAYQIPEIALR.A(30)<br>K.YFWLGDAK.A(36)
461	Putative signal peptide	gi|126641049	3	R.GDASRPVYGVTAQR.A(38)<br>R.LTAQPLPYLELGASR.T(45)<br>K.GNDNVYGDTENPSNQLAGFDGR.L (46)
462	F0F1 ATP synthase subunit alpha	gi|162286757	6	R.VLEVPVGPELLGR.V(33)<br>R.QSVDQPVQTGYK.S(28)<br>K.SVDTMIPVGR.G(27)<br>R.EAYPGDVFYLHSR.L(64)<br>R.ELAAFAQFASDLDEATR.K(43)<br>K.AGIESFKATQTY.-(30)
463	Putative protein (DcaP-like)	gi|126642757	5	R.QVQQQQQQVQQQQQVQLAEVK.A(19)<br>R.LGLDFNTPVGDDK.V(38)<br>K.IEVDFAGSTTDSNGSLR.I(38)<br>K.ITQGYAEGR.G(35)<br>R.STLAYGAQFSDDGTDYAR.L(64)
464	Putative protein (DcaP-like)	gi|126642784	9	R.STLAYGAQFSDDGTDYAR.L(88)<br>K.AQPQPVAAPVSPLAGFK.S(105)<br>K.SKAGADVNLYGFVR.G(88)<br>K.AGADVNLYGFVR.G(112)<br>K.IEVDFAGSTTDSNGSLR.I(58)<br>K.LGPTTQLFVSAEKGDSTTSVTGDSIK.Y(41)<br>K.ITQGYAEGR.G(67)<br>K.SQLADDDKTGWGVAVGTDFK.V(44)<br>R.STLAYGAQFSDDGTDYAR.L(141)
465	Putative Omp (OprD)	gi|126640296	5	K.GFVEDANGSILFR.T(53)<br>K.NAGNNMIPQHNDGSAYDHWAR.G(28)<br>R.YGTQVLDLPVLASNTAR.L(92)<br>K.NQYSDQIATDQNGLDR.A(59)<br>K.TSNGADDSNESEFFNQVK.Y(49)

Table S3. Continued.

Spot No.	Identified protein	Accession no.	Matched peptides	Matched peptides (Ion score)
466	Putative glucose-sensitive porin (OprB-like)	gi|126642873	9	K.ILGWQDTEAQITLTYR.D(33)<br>R.DGQSLSEHSPALAGHQSSVQEVWGR.E(93)<br>R.VKYNLQPDLYTQVGVYEYNPENLER.G(41)<br>K.YNLQPDLYTQVGVYEYNPENLER.G(100)<br>K.GFNLSTDGSHGAIIPAEVVWSPK.L(41)<br>K.LGVQSMPGEYR.L(54)<br>K.LFQPADQTDR.G(28)<br>K.GLLNQRPQDELALGVAR.I(49)<br>R.IHINDDWSDVQAK.E(110)
467	Omp38 precursor (OmpA)	gi|126642864	9	K.IKPYVLLGAGHYK.Y (50)<br>K.YDFDGVNR.G (42)<br>R.GTRGTSEEGTLGNAGVGAFWR.L (13)<br>R.GTSEEGTLGNAGVGAFWR.L (51)<br>K.VAEKLSEYPNATAR.I (72)<br>R.IEGHTDNTGPR.K (27)<br>K.SALVNEYNVDASR.L (47)<br>R.LSTQGFAWDQPIADNKTK.E (31)<br>R.VFATITGSR.T (36)
468	Omp 33-36 kDa	gi|193078641	5	K.GEAYVPTPYLPVYASATYNHTDVDGK.N (102)<br>K.LSVGATFVGNDGEADIKDNNLGEFR.Q (219)<br>K.DNNLGEFR.Q (67)<br>K.KSSYDTQTIGLNAK.F (107)<br>K.SSYDTQTIGLNAK.F (133)
469	Omp CarO precursor	gi|126642573	3	R.WAQGLYVAAGAAYLDNDYDLTKR.S (46)<br>K.QGTFVNAAGGNADADLR.A (69)<br>K.QGTFVNAAGGNADADLRAEENK.I (30)
470	Putative Omp (Omp 25)	gi 72535027	8	-.YQAEVGGSYNYLDPDNGSSVSK.F(31)<br>K.FGVDGTYYFNPVQTR.N(29)<br>R.NAPLAEAAFLNR.A(86)<br>K.DTQYGVGVEYFVPNSDFYLSGDVGR.N(104)<br>K.TLSLGVDYYNNDLTDKDEFGINAK.K(32)<br>K.KFLNQQVSVEGR.V(40)<br>K.FLNQQVSVEGR.V(74)<br>R.VGFGDNDNTYGVR.A(83)
471	Putative Omp	gi|126643324	7	K.FGVDGTYYFNPVQTR.N(70)<br>R.NAPLAEAAFLNR.A(91)<br>K.DTQYGVGVEYFVPNSDFYLSGDVGR.N(131)<br>K.YVTQVGQHDVNLEAYGAFGDLDEYKVR.G(31)<br>K.KFLNQQVSVEGR.V(52)<br>K.FLNQQVSVEGR.V(70)<br>R.VGFGDNDNTYGVR.A(62)
472	Omp CarO precursor	gi|126642573	4	R.WAQGLYVAAGAAYLDNDYDLTKR.S (117)<br>K.NDIAPYLGFGFAPK.I (81)<br>K.INKNWGVFGEVGAYYTGNPTVELDK.Q (30)<br>K.QGTFVNAAGGNADADLR.A (80)
473	Omp W	gi|126640380	5	R.FTPYIGIGATAFIPWDEQGVADK.V(54)<br>R.FTPYIGIGATAFIPWDEQGVADKVK.E(47)<br>K.EDFGVAGQIGFNFQPADAK.N(80)<br>K.NWGVFVDVR.Y(56<br>K.FDLDINPFVYTLGYSYKF.-

Table S3. Continued.

Spot No.	Identified protein	Accession no.	Matched peptides	Matched peptides (Ion score)
474	Omp W	gi126640380	6	K.FGGSVIAPSEDTTTALGVVK.A(94)
				K.YHFKNSTR.F(27)
				R.FTPYIGIGATAFIPWDEQGVADK.V(51)
				R.FTPYIGIGATAFIPWDEQGVADK.V(37)
				K.EDFGVAGQIGFNFQPADAK.N(76)
				K.NWGVFVDVR.Y(70)
475	Putative peptidoglycan-binding LysM	gi|193076594	4	K.NTAPAEPQAAPATPAEPSAQEIANK.L (24)
				K.IAKEFYGDANQYQK.I (54)
				K.EFYGDANQYQK.I (57)
				K.DPDEIFPGQVLR.I (28)
476	Putative peptidoglycan-binding LysM	gi|126640876	3	K.IAKEFYGDANQYQK.I (48)
				K.EFYGDANQYQK.I (45)
				K.DPDEIFPGQVLR.I (28)
478	Glutathione peroxidase	gi|126640260	2	K.CGLTPQYEGLEK.L (49)
				R.FAPNLTADDEQIVK.A (49)
480	Putative protease	gi|126642815	10	M.VPFQFLTGLGYTVHAVCPNKK.N
				K.NGEHIATAIHDFEGEQTYSEK.R
				K.NGEHIATAIHDFEGEQTYSEKR.G
				K.NGEHIATAIHDFEGEQTYSEKR.G
				R.GHNFAINYDFDAINTEDYVGLVIPGGR.A
				R.MNERVVEIVR.E
				R.VVEIVREFDR.V
				R.VKKPIAAVCHGAQLLAAADVLK.D
				R.LCSAYPACAAEVK.L
				R.LCSAYPACAAEVK.L
481	Bacterioferritin	gi|126640856	4	R.DQYLIHSR.M (38)
				R.MYEDWGLNK.I (36)
				R.IDHEMQEEASHADAIIR.R (26)
				K.ADLALEYHVR.E (50)
483*	F0F1 ATP synthase β-subunit	gi|162286755	14	K.IYDALQVDGTETTLEVQQQLGDGVVR.T
				R.GLTVTSTNAPISVPVGTATLGR.I
				R.IMDVLGRPIDEAGPVATEER.L
				K.VGLFGGAGVGK.T
				K.AHSGLSVFAGVGER.T
				R.DVLLFVDNIYR.Y
				R.YTLAGTEVSALLGR.M
				R.MPSAVGYQPTLAEEMGVLQER.I
				R.MPSAVGYQPTLAEEMGVLQER.I
				R.DIASSGIYPAIDPLDSTSR.Q
				R.QLDPLVVGQEHYEIAR.A
				R.AVQNVLQR.Y
				K.DIIAILGMDELAEEDK.L
				K.LVPLKETIR.G
484	F0F1 ATP synthase β-subunit	gi|162286755	7	R.VLEVPVGPELLGR.V (48)
				R.DRGEDALIIYDDLSK.Q (44)
				R.EAYPGDVFYLHSR.L (78)
				R.VSAEYVEKFTNGAVTGK.T (67)
				R.ELAAFAQFASDLDEATR.K (48)
				R.ELAAFAQFASDLDEATRK.Q (41)
				K.QIDETGDYNKDIEAAIK.A (66)

Table S3. Continued.

Spot No.	Identified protein	Accession no.	Matched peptides	Matched peptides (Ion score)
485	F0F1 ATP synthase α-subunit	gi|162286757	8	K.NEGTIVMVSDGIVR.I (55)<br>R.VLEVPVGPELLGR.V (62)<br>R.VVDALGNPIDGKGPIDAK.L (110)<br>R.DRGEDALIIYDDLSK.Q (41)<br>R.EAYPGDVFYLHSR.L (70)<br>R.VSAEYVEKFTNGAVTGK.T (60)<br>R.ELAAFAQFASDLDEATR.K (38)<br>K.QIDETGDYNKDIEAAIK.A (88)
487	Putative protein (DcaP-like)	gi|126642784	2	K.ITQGYAEGR.G (59)<br>R.STLAYGAQFSDDGTDYAR.L (50)
488	Putative protein (DcaP-like)	gi|126642784	4	R.QVQQQQQQVQQQQQVQLAEVK.A (103)<br>K.IEVDFAGSTTDSNGSLR.I (59)<br>K.ITQGYAEGR.G (35)<br>R.STLAYGAQFSDDGTDYAR.L (113)
489	Elongation factor Tu	gi|162286746	3	R.GITINTSHVEYDSPTR.H (33)<br>R.ELLSTYDFPGDDTPVIR.G (31)<br>K.FDAEVYVLSKEEGGR.H (53)
490	F0F1 ATP synthase γ-subunit	gi|126640257	1	K.FDAEVYVLSKEEGGR.H
491	Aspartate carbamoyltransferase catalytic subunit	gi|162286738	4	R.LSANVLNIDIAR.S (44)<br>K.VAIINAGDGR.H (39)<br>R.SDVAALQTLGCKDIR.V (32)<br>R.IDSPALSSQSEFYR.M (25)
492	Elongation factor Ts	gi|126642362	5	K.ALTEANGDIELAIDNLR.K (98)<br>K.IGENIQVR.R (41)<br>K.YLNEVALDR.Q (38)<br>K.ATGTNVANFVR.F (21)<br>K.AELSFAEEVAAAQAAAK.- (109)
493	Two-component response regulator	gi|193078590	2	M.SLVVPAEHPETVHNETDRVER.I (28)<br>R.ILVVDDDVRLR.T (36)
494	Hypothetical protein A1S_0015	gi|126640130	3	K.LGFEYNAR.L (30)<br>R.LLANDIQPQHR.E (52)<br>R.GEGVEQSFTPDVGIR.H (44)
495	NADH dehydrogenase I chain B	gi|193076535	3	R.ANPDADQYPLQDR.Q (41)<br>R.LYEQMLEPK.W (59)<br>R.LYEQMLEPK.W (29)
496	Omp38 precursor (OmpA)	gi|126642864*	2	K.VAEKLSEYPNATAR.I (29)<br>K.SALVNEYNVDASR.L (50)
497	50S ribosomal protein L10	gi|193076142	3	M.ALLIEDKK.Q (49)<br>R.LFEEFAKTNK.A (71)<br>K.VYQGADVSVIANLPNQEK.A (69)
498	Universal stress protein	gi|126642117	2	K.ALCAEEGVDIETQIIQGEISSDGILK.A (61)<br>K.AVEELGSDLIVIGSHGR.K (64)
499	F0F1 ATP synthase γ-subunit	gi|126640257*	1	R.ADNLDEAAILEAR.K (61)

All the proteins were identified from MASCOT files with NCBInr. http://www.matrixscience.com/
(*) Identified by MALDI-TOF analyses.
